# Chemical Isotope Labeling and Dual-Filtering Strategy for Comprehensive Profiling of Urinary Glucuronide Conjugates

**DOI:** 10.1021/acs.analchem.4c02339

**Published:** 2024-08-05

**Authors:** Zhi-Qiang Chen, Ru-Jie Yang, Chao-Wei Zhu, Yang Li, Ru Yan, Jian-Bo Wan

**Affiliations:** † State Key Laboratory of Quality Research in Chinese Medicine, Institute of Chinese Medical Sciences, University of Macau, Taipa Macao SAR, China; ‡ Shenzhen People’s Hospital, Shenzhen, Guangdong 518000, China

## Abstract

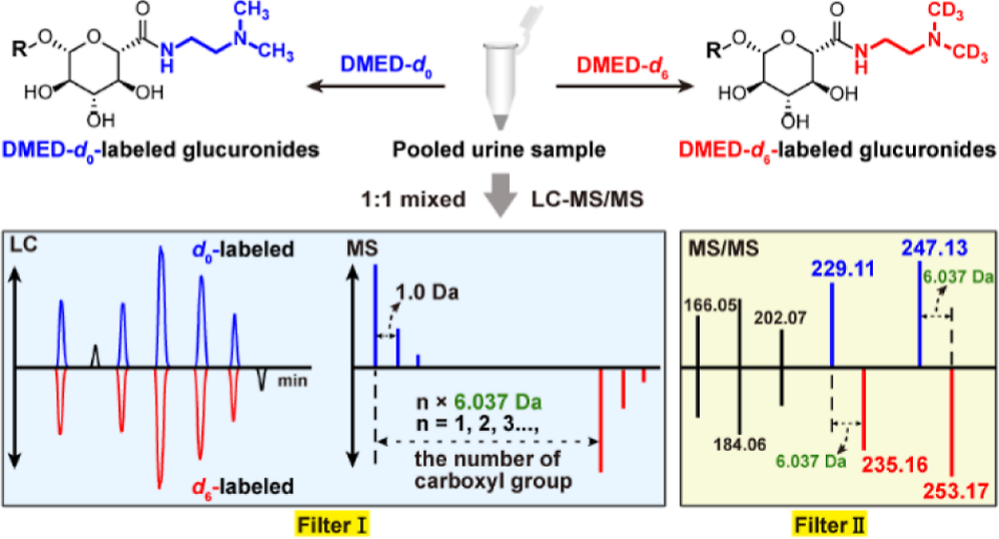

Glucuronidation, a crucial process in phase II metabolism, plays a vital role in the detoxification and elimination of endogenous substances and xenobiotics. A comprehensive and confident profiling of glucuronate-conjugated metabolites is imperative to understanding their roles in physiological and pathological processes. In this study, a chemical isotope labeling and dual-filtering strategy was developed for global profiling of glucuronide metabolites in biological samples. *N*,*N*-Dimethyl ethylenediamine (DMED-*d*_0_) and its deuterated counterpart DMED-*d*_6_ were used to label carboxylic acids through an amidation reaction. First, carboxyl-containing compounds were extracted based on a characteristic mass difference (Δ*m*/*z*, 6.037 Da) observed in MS between light- and heavy-labeled metabolites (filter I). Subsequently, within the pool of carboxyl-containing compounds, glucuronides were identified using two pairs of diagnostic ions (*m*/*z* 247.1294/253.1665 and 229.1188/235.1559 for DMED-*d*_0_/DMED-*d*_6_-labeled glucuronides) originating from the fragmentation of the derivatized glucuronic acid group in MS/MS (filter II). Compared with non-derivatization, DEMD labeling significantly enhanced the detection sensitivity of glucuronides, as evidenced by a 3- to 55-fold decrease in limits of detection for representative standards. The strategy was applied to profiling glucuronide metabolites in urine samples from colorectal cancer (CRC) patients. A total of 685 features were screened as potential glucuronides, among which 181 were annotated, mainly including glucuronides derived from lipids, organic oxygen, and phenylpropanoids. Enzymatic biosynthesis was employed to accurately identify unknown glucuronides without standards, demonstrating the reliability of the dual-filtering strategy. Our strategy exhibits great potential for profiling the glucuronide metabolome with high coverage and confidence to reveal changes in CRC and other diseases.

Glucuronidation is one of the dominant metabolic pathways involved in the elimination of various endogenous and xenobiotic compounds and plays a crucial role in metabolic homeostasis and xenobiotic disposition. The glucuronidated metabolites were generally considered to be inactive. Thus, glucuronidation has been widely accepted as a detoxification mechanism.^1^ In recent decades, glucuronide conjugates have attracted more attention due to increasing reports on their biological activities, both toxic and pharmacological.^2−4^ Glucuronidation homeostasis of endogenous compounds and the elimination of xenobiotics via glucuronidation are coordinately regulated by host UDP-glucuronosyltransferases (UGTs) and β-glucuronidase enzymes (GUSs) widely distributed in the gut microbiota. Alterations in the expression and activity of UGTs and/or GUSs under pathological conditions or medication might disrupt glucuronidation homeostasis, complicating health problems or leading to unexpected clinical outcomes.^5^ Furthermore, glucuronide conjugates of several endogenous or exogenous compounds have been reported as potential biomarkers for specific conditions.^6^ For instance, 27-nor-5β-cholestane-3,7,12,24,25 pentol glucuronide, *n*-acetyltyramine-*O*-glucuronide, and cholestanetetrol glucuronide were reported as biomarkers for epithelium ovarian cancer,^7^ onchocerciasis,^8^ and cerebrotendinous xanthomatosis,^9^ respectively. Thus, it is imperative to establish a method that offers comprehensive and confident profiling of glucuronate-conjugated metabolites to promote mechanistic understanding of their roles in physiological, pathological, or pharmacological processes.

The biotransformation of structurally diverse aglycones gives rise to glucuronate-conjugated metabolites with varied physicochemical properties,^10,11^ posing great challenges in simultaneously profiling these metabolites.^12^ The characteristic neutral loss of 176.0321 Da corresponding to one glucuronic acid molecule has been widely utilized for glucuronide profiling by liquid chromatography-tandem mass spectrometry (LC–MS).^13,14^ Although the detection with neutral loss offers several advantages, such as convenient and rapid analysis without additional derivatization or hydrolysis treatment, it is challenging to exclude false positives based solely on a single screening condition. Recently, a strategy combining post-deconvolution MS/MS spectra extraction with data-independent acquisition (PDMS2E-DIA) was developed to profile urinary glucuronate-conjugated metabolome by incorporating an enzymatic hydrolysis and an algorithm that filters out the unconjugated and conjugated metabolite ion pairs and reconstructs MS/MS spectra from DIA analysis.^15^ This strategy can enhance identification scores of potential metabolites that can be conjugated by glucuronate through the removal of noise peaks. In the past decades, chemical derivatization-assisted LC–MS strategies have been widely developed for metabolome analysis with diverse advantages, such as improving the analytical performance and offering more characteristic structural information.^16−19^ This chemical derivatization strategy has been applied to accurately quantify specific glucuronides^20^ or differentiation of isomeric acyl-, *N*-, and *O*-glucuronide derivatives of drugs.^21,22^ These studies only focused on small numbers of specific glucuronides, such as drug metabolites or a particular type of glucuronides.

In this study, a chemical isotope labeling and dual-filtering strategy was proposed to comprehensively profile glucuronate-conjugated metabolites in biological samples with high coverage and confidence. The glucuronidated metabolites were derivatized with a pair of isotope probes, *N*,*N*-dimethyl ethylenediamine (DMED-*d*_0_) and DMED-*d*_6_, both specifically targeting the carboxylic group of glucuronic acid, regardless of the types of the glycosidic bond (acyl-, *O*-, and *N*-glucuronides). Carboxyl-containing compounds were first extracted based on similar retention times (RT) in LC chromatogram and a characteristic mass difference in MS spectra between light- and heavy-labeled metabolites. Then, glucuronide metabolites were identified from the pool of carboxyl-containing compounds using two pairs of diagnostic ions (*m*/*z* 247.1294/253.1665 and 229.1188/235.1559 for *d*_0_/*d*_6_-labeled glucuronides) in the MS/MS spectra. As a proof of concept, the newly developed method was applied to profile glucuronide submetabolome in urine samples from CRC patients.

## Materials and Methods

### Chemicals and Reagents

Fifteen glucuronide standards were purchased from Sigma-Aldrich (St. Louis, MO, USA), Yuanye Bio-Technology Co. (Shanghai, China), and ZZBIO Co. (Shanghai, China). Detailed information and chemical structures of glucuronide standards are shown in Table 1 and Figure S1. *O*-(7-Azabenzotriazol-1-yl)-*N*,*N*,*N*′,*N*′-tetramethyluronium hexafluorophosphate (HATU), triethylamine (TEA), and DMED were provided by Sigma-Aldrich (St. Louis, MO, USA). DMED-*d*_6_ was chemically synthesized by WuXi AppTec Limited (Hongkong, China) and identified by LC–MS and NMR spectra (Figure S2). LC–MS grade acetonitrile (ACN) was supplied by Merk (Darmstadt, Germany). Ultrapure water was purified by a Milli-Q system (Millipore, Bedford, MA).

**Table 1:** Detailed Information on 15 Glucuronide Standards and Their DMED Derivatives

no.	glucuronide	number of carboxyl groups	glycosidic bond	number of glucuronic acid moiety	non-labeled			DMED-labeled (nM)			LOD^a^ (nM)	LOD^b^ (nM)	fold of LOD decrease
					formula	accurate mass	RT(min)	formula	accurate mass	RT (min)			
1	4-nitrophenyl glucuronide	1	*O*-	Mono-	C_12_H_13_NO_9_	315.059	5.38	C_16_H_23_N_3_O_8_	385.1485	6.33	12.49	0.33	38
2	8-hydroxyquinoline glucuronide	1	*O*-	Mono-	C_15_H_15_NO_7_	321.0849	5.36	C_19_H_25_N_3_O_6_	391.1743	6.81	20.23	0.51	39
3	4-methylumbelliferyl glucuronide	1	*O*-	Mono-	C_16_H_16_O_9_	352.0794	6.72	C_20_H_26_N_2_O_8_	422.1689	7.16	10.34	0.77	13
4	melatonin glucuronide	1	*N*-	Mono-	C_19_H_24_N_2_O_8_	408.1533	7.02	C_23_H_34_N_4_O_7_	478.2427	7.30	9.40	0.24	40
5	baicalin	1	*O*-	Mono-	C_21_H_18_O_11_	446.0849	10.67	C_25_H_28_N_2_O_10_	516.1744	7.30	17.12	5.63	3
6	*p*-cresol glucuronide	1	*O*-	Mono-	C_13_H_16_O_7_	284.0896	7.10	C_17_H_26_N_2_O_6_	354.1791	7.46	12.65	0.63	20
7	thyroxine glucuronide	2	*O*-	Mono-	C_21_H_19_I_4_NO_10_	952.7188	11.03	C_29_H_39_I_4_N_5_O_8_	1092.8977	9.20	27.12	9.11	3
8	glycyrrhizin	3	*O*-	Di-	C_42_H_62_O_16_	822.4038	16.14	C_54_H_92_N_6_O_13_	1032.6722	10.79	5.29	1.03	5
9	phenolphthalein glucuronide	1	*O*-	Mono-	C_26_H_22_O_10_	494.1213	11.32	C_30_H_32_N_2_O_9_	564.2108	11.21	5.08	1.20	4
10	estradiol 3-glucuronide	1	*O*-	Mono-	C_24_H_32_O_8_	448.2097	11.25	C_28_H_42_N_2_O_7_	518.2992	11.22	2.67	0.23	12
11	oroxylin A 7-*O*-glucuronide	1	*O*-	Mono-	C_22_H_20_O_11_	460.1006	11.63	C_26_H_30_N_2_O_10_	530.1900	11.68	11.06	1.47	8
12	wogonoside	1	*O*-	Mono-	C_22_H_20_O_11_	460.1006	12.19	C_26_H_30_N_2_O_10_	530.1900	12.09	8.96	0.85	10
13	chenodeoxycholic acid 3-glucuronide	2	*O*-	Mono-	C_30_H_48_O_10_	568.3247	18.89	C_38_H_68_N_4_O_8_	708.5037	12.63	3.13	0.29	11
14	diethylstibestrol glucuronide	1	*O*-	Mono-	C_24_H_28_O_8_	444.1784	13.13	C_28_H_38_N_2_O_7_	514.2679	12.58	1.48	0.30	5
15	androsterone glucuronide	1	*O*-	Mono-	C_25_H_38_O_8_	466.2567	15.16	C_29_H_48_N_2_O_7_	536.3462	14.20	11.24	0.21	55

a LODs of glucuronides detected by non-derivatization method.

b LODs of glucuronides detected by derivatization method. RT, retention time; LOD, limit of detection.

### Biosynthesis of Hyodeoxycholic acid Glucuronide

Hyodeoxycholic acid (HDCA) glucuronide, a commercially unavailable metabolite, was biosynthesized through an *in vitro* glucuronidation reaction of HDCA in human liver microsomes (purchased from Sigma-Aldrich (St. Louis, MO, USA)). Briefly, the incubation system contained 0.1 mM HDCA, 8 mM MgCl_2_, 25 μg/mL alamethicin, and 1 mg/mL human liver microsomes in Tris–HCL buffer (50 mM pH 7.4) in a total volume of 0.1 mL. The reaction was initiated by adding UDPGA (20 mM, 10 μL) and maintained at 37 °C for 6 h. The reaction was terminated by adding 0.1 mL of ice-cold ACN. After centrifugation at 15,000*g*, 4 °C for 10 min, the resulting supernatant was collected and subjected to LC–MS analysis.

### Urine Sample Collection and Preparation

Urine samples were collected from twenty patients who were diagnosed by Shenzhen People’s Hospital (Shenzhen, China) as early (1 polyp, 1 hyperplasia, 1 fibroid, and 6 adenomas) or 11 advanced CRC (10 stage III and 1 stage IV). The clinical and biochemical characteristics of CRC patients were summarized in Table S1. Urine samples were stored at −80 °C until analysis. The study protocol was approved by the ethics committee of Shenzhen People’s Hospital as minimal risk research (no invasion or intervention). Written informed consent was acquired from each patient before sample collection. A quality control (QC) sample was prepared by mixing an equal volume of urine samples from each individual. An aliquot (100 μL) of the pooled sample was mixed with ACN (1:1, v/v) and vortexed. After centrifugation at 15,000*g*, 4 °C for 15 min, the supernatant was evaporated to dryness under a stream of nitrogen, and the residue was subjected to further derivatization.

### Derivatization of Glucuronides with DMED

DMED-*d*_0_ and DMED-*d*_6_ solutions (10 mM) were diluted with ACN. The prepared QC samples (10 μL) or the mixture of 15 glucuronide standards (100 μM each) were separately mixed with 10 μL of DMED-*d*_0_ and DMED-*d*_6_ derivatization solutions and sequentially added with 10 μL of HATU (50 μM, dissolved in ACN) and 10 μL of TEA (200 μM, dissolved in ACN). The mixture was incubated at room temperature for 10 min, evaporated under a nitrogen stream, and redissolved in 50 μL of ACN. For urinary glucuronide profiling, the DMED-*d*_0_-labeled pooled sample was mixed with an equal mole of DMED-*d*_6_-labeled pooled sample, subsequently detected by LC–MS. For the discovery of differentiated glucuronides in urine samples from CRC patients, individual samples were derivatized by DMED-*d*_0_.

### UPLC-HRMS/MS Analysis

The derivatized glucuronides were analyzed by an ultraperformance liquid chromatography (UPLC) system coupled with a SYNAPT G2-Si QTOF mass spectrometry (Waters Corp., MA, USA) equipped with an electrospray ionization (ESI) source. Chromatographic separation was conducted on an ACQUITY UPLC HSS T3 column (100 × 2.1 mm i.d., 1.8 μm) maintained at a temperature of 40 °C. A gradient elution was employed with a mobile phase comprising of ammonium formate aqueous solution (10 mM, pH 3.8, A) and acetonitrile (B) at a flow rate of 0.3 mL/min as follows: 5% B for 0–2 min; 5–35% B in 2–12 min; 35% B in 12–16 min; 35–75% B in 16–25 min; 75–95% B in 25–26 min; 95% B in 26–27 min; 95–5% B in 27–28 min, and then 2 min of re-equilibration at 5% B. The injection volume was 1 μL. The analytes were detected by QTOF MS in a positive MS^E^ mode. MS data were acquired in continuum mode within the *m*/*z* range of 100–1200. Other main parameters for MS analysis were configured as follows: capillary voltage, 2.5 kV; cone voltage (CV), 30 V; source temperature, 120 °C; desolvation gas temperature, 450 °C; cone gas flow, 50 L/h; and desolvation gas, 600 L/h.

### Data Processing

The raw data was acquired by MassLynx software (version 4.2, Waters Corp., MA, USA) and processed with Progenesis QI software (version 2.4, Waters Corp., MA, USA) for alignment, peak picking, and exporting a feature list. Orthogonal partial least-squares-discriminant analysis (OPLS-DA) of urinary glucuronides in CRC patients with early and advanced stages was conducted by MetaboAnalyst version 5.0 (https://www.metaboanalyst.ca/). Features were considered the differentiated glucuronides between the groups, which satisfied the criteria of fold change (FC) > 2, variable importance in projection (VIP) > 1, and *P* value < 0.05.

## Results and Discussion

### Group Classification of Glucuronides

To facilitate the profiling and structural identification of glucuronides, a comprehensive search of currently available information was conducted using “glucuronide,” “glucuronic acid,” and “glucopyranosiduronic acid” as keywords in the HMDB and PubChem databases as of 16th April 2024. After manually deduping and merging, a total of 3880 records of glucuronide compounds accounting for 1.6% of HMDB and 0.3% of PubChem were retrieved. Notably, endogenous glucuronides constituted only a minor portion (448/3,880, 12%) of the recorded glucuronides (Figure S3). Most endogenous glucuronides belong to the lipids and lipid-like molecules group, accounting for 81% of the total (365/448). Within the lipids and lipid-like molecules, steroid conjugates, particularly sex hormone glucuronides (i.e., estrogens and androgens) and bile acid glucuronides, constitute the predominant portion (338/365, 93%). The plausible explanation is that the sex-related hormone glucuronides have been extensively studied due to their crucial physiological roles.^23^ The detection of other types of endogenous glucuronides may still be challenging due to their low abundance or complex physicochemical properties.

### Optimization of Derivatization Conditions

**Figure 1:**
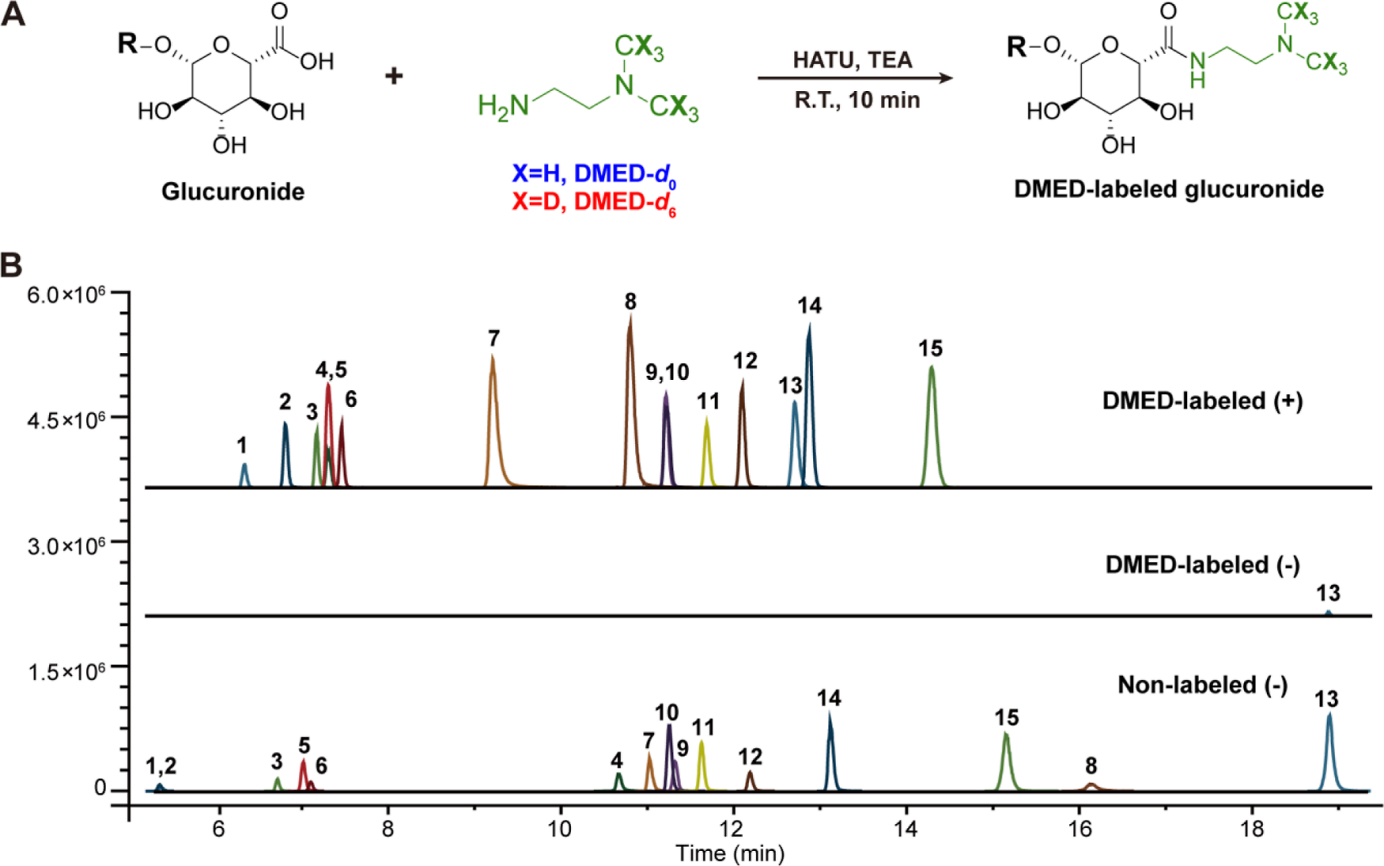
(A) Derivatization reaction of glucuronate-conjugated metabolites with DMED-*d*_0_/*d*_6_. (B) LC–MS chromatograms of 15 derivatized (up, positive) and nonderivatized glucuronides (middle, negative) in the DMED-labeled standard sample and chromatograms of nonderivatized glucuronides (bottom, negative). DMED, *N*,*N*-dimethyl ethylenediamine; HATU, 2-(7-azabenzotriazol-1-yl)-*N*,*N*, *N*′, *N*′-tetramethyluronium hexafluorophosphate; TEA, triethylamine; and R.T., room temperature. The glucuronides is numbered the same as shown in Table 1.

DMED has been widely utilized as a derivatization agent with high reactivity to profile carboxyl-containing metabolites^24−27^ and fatty acid esters of hydroxy fatty acids^28^ in various biological samples. The derivatization reactions are simple and rapid with few side reaction products and high yield.^29^ In this study, DMED-*d*_0_ and its deuterated counterpart DMED-*d*_6_ were first used to label glucuronate-conjugated metabolites, specifically targeting the carboxylic group of the glucuronic acid moiety regardless of the glycosidic bond type (Figure 1A). To ensure the optimal reaction efficiency, the amidation conditions, including reaction temperature (20–50 °C), reaction duration (10–120 min), and the ratio of DMED to substrate (10:1–1000:1), were further optimized using glucuronide standards in a single-factor experiment. As depicted in Figure S4, the peak areas of most investigated glucuronides reached their maximum values at 20 °C, 10 min of duration, and 100:1 of DMED/substrate ratio.

Using the optimized reaction conditions, 15 glucuronide standards were labeled with DMED and detected by LC–MS. The non-derivatized glucuronides were analyzed in negative ion mode to compare the sensitivity (Figure 1B). Upon DMED labeling, the detection sensitivities of glucuronides were increased significantly, as evidenced by 3- to 55-fold decreases in the limits of detection (LODs) of glucuronide standards (Table 1). Incorporating the tertiary amine group with a high proton affinity under acidic conditions substantially improved the ionization efficiency of DMED-labeled glucuronides, leading to a notable increase in MS detection sensitivity and facilitating the detection of glucuronides in low abundance. Due to the high reactivity of DMED toward the carboxylic group of glucuronides under the catalysis of HATU in a weak basic environment provided by TEA, after the derivatization of 15 mixed standards, only trace amount of the non-derivatized standard (chenodeoxycholic acid 3-glucuronide, 13) was detected in negative ion mode (Figure 1B, middle), indicating that most glucuronides have been entirely labeled by DMED. These data suggest the high derivatization efficiency of DMED toward glucuronides.

### Optimization of LC–MS Conditions

Mobile phase modifiers are recognized to improve peak resolution in the chromatogram and ionization capacity.^30^ In this study, we compared the addition of formic acid and ammonium formate as the common modifiers of the mobile phases on the detection sensitivity of four typical glucuronides, which represent steroid, flavonoid, heteroatom, and other common glucuronide core types. These four DMED-labeled glucuronides exhibited significantly higher intensities with ammonium formate as a modifier when compared to those obtained with formic acid (Figure S5), suggesting that ammonium formate facilitates the formation of protonated ions during electrospray ionization. The underlying mechanisms may involve molecular interactions between ammonium ion and DMED-labeled glucuronides, pH modification, and ionic strength, resulting in the enhanced protonation and subsequent ionization in the gas phase during electrospray ionization.

We also observed an influence of the injection volume on the chromatographic separation of DMED-labeled glucuronides. When 3 μL of injection volume was used, extracted ion chromatograms (EICs) of four glucuronides, namely, *p*-cresol glucuronide, 8-hydroxyquinoline glucuronide, 4-nitrophenyl glucuronide, and 4-methylumbelliferyl glucuronide, displayed one bump before a slightly fronting peak, of which the intensities were proportional to the analyte concentration. The bumps disappeared when the analytes were injected separately or as a mixture at 1 μL, regardless of the concentration or the storage time of the prepared solutions, excluding the reasons of column overload or degradation during storage (Figure S6). These findings indicate that the DMED-labeled glucuronides are more sensitive to sample dispersion and peak broadening caused by higher injection volumes. A plausible explanation for this observation is that a smaller injection volume may lead to improved sample dispersion and reduced peak broadening, ultimately resulting in a single, well-defined peak in the chromatogram. However, further investigation is warranted to gain a comprehensive understanding of the causes of this phenomenon.

The CV and collision energy (CE) values were further optimized by using glucuronide standards. As depicted in Figure S7, a CV of 30 V and a CE value ranging from 10 to 30 eV appeared to be more suitable conditions to improve the detection of the analytes and the acquisition of feature fragments in the MS^E^ mode, facilitating screening and structural elucidation of glucuronate-conjugated metabolites in the biological samples.

### Specific Fragmentation Patterns of DMED-Labeled Glucuronides

**Figure 2:**
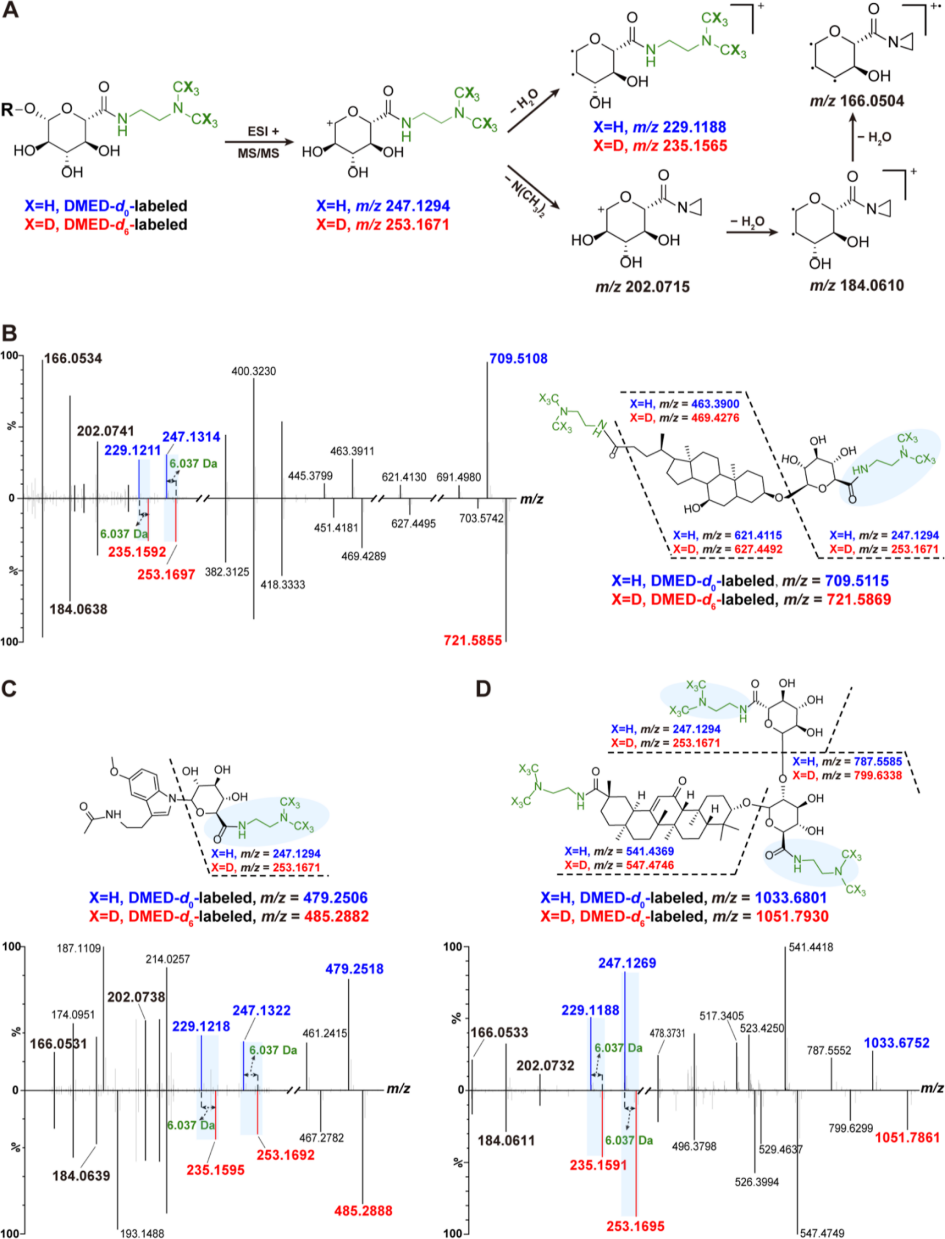
LC–MS characteristics of DMED-labeled glucuronides. (A) Potential fragmentation pattern of DMED-labeled glucuronides. Chemical structure and MS/MS spectra of representative DMED-*d*_0_ (blue) and DMED-*d*_6_ (red) labeled glucuronides, including (B) chenodeoxycholic acid 3-glucuronide, (C) melatonin glucuronide, and (D) glycyrrhizin.

We utilized 15 glucuronide standards to investigate the fragmentation patterns of DMED-*d*_0_/*d*_6_-labeled glucuronides. As depicted in Figure 2A, all labeled glucuronides displayed similar fragmentation pathways in their MS/MS spectra. Specifically, two pairs of diagnostic fragments generated from DMED-*d*_0_/*d*_6_-labeled glucuronides displayed an *m*/*z* difference of 6.037 Da in the MS/MS spectra due to the presence of isotopic tags, including the first one (*m*/*z* 247.1294/253.1665) resulted from the loss of deconjugated aglycone and subsequent dehydration leading to the generation of another pair of fragment ions (*m*/*z* 229.1188/235.1559). Furthermore, DMED-*d*_0_/*d*_6_-labeled glucuronides shared three common characteristic fragments at *m*/*z* 202.0715, 184.0610, and 166.0504, corresponding to a further neutral loss of the N(CH_3_)_2_ group and sequential dehydration. The two pairs of diagnostic fragments and the three characteristic common fragments were observed in all of the DMED-*d*_0_/*d*_6_-labeled glucuronides. The specific fragmentation patterns of glucuronides were illustrated with the labeled derivatives of CDCA-3-G (a mono-*O*-glucuronide with two carboxyl groups, Figure 2B), melatonin glucuronide (a mono-*N*-glucuronide with one carboxyl group, Figure 2C), and glycyrrhizin (a di-*O*-glucuronide with three carboxyl groups, Figure 2D). Taking CDCA-3-G as an example, the precursor ions of DMED-*d*_0_/*d*_6_-labeled CDCA-3-G generated fragment ions at *m*/*z* 247.1294/253.1665 and *m*/*z* 229.1188/235.1559, corresponding to the cleavage of the glycosidic bond and further dehydration (Figure 2B). Similar fragmentation patterns were also observed in MS/MS spectra of melatonin glucuronide and glycyrrhizin derivatives (Figure 2C,D). Collectively, DMED-*d*_0_/*d*_6_-labeled glucuronides exhibit the ability to produce two pairs of diagnostic fragments (*m*/*z* 247.1294/253.1665 and *m*/*z* 229.1188/235.1559 for *d*_0_/*d*_6_-labeled), thereby facilitate the identification of glucuronide metabolites in biological samples.

**Figure 3:**
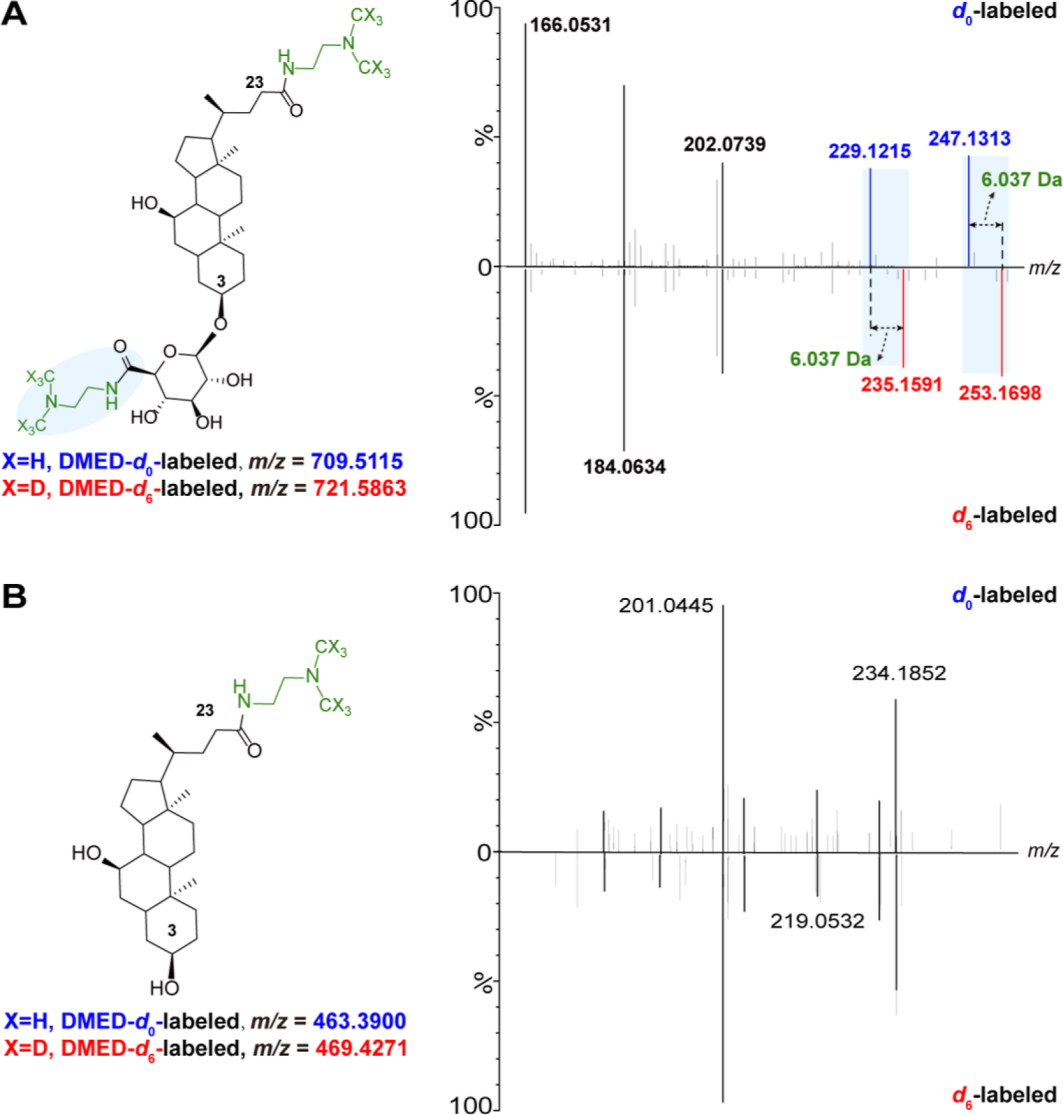
Specific fragmentation patterns of DMED-*d*_0_/*d*_6_-labeled glucuronides. Chemical structure and MS/MS spectra of DMED-labeled (A) chenodeoxycholic acid 3-glucuronide and (B) chenodeoxycholic acid.

DMED is a specific labeling agent for the carboxylic group, including the carboxyl group of glucuronic acid. This means that any metabolite containing a carboxyl group can be derivatized using DMED. To investigate the uniqueness of the observed diagnostic fragments in labeled glucuronides, the MS/MS spectra of CDCA (a primary bile acid with one carboxyl group at C-23 and one glucuronic acid group at C-3) and its monoglucuronide CDCA-3-G were compared (Figure 3). The C-23 carboxyl and C-3 glucuronic acid groups in CDCA-3-G were derivatized with DMED. The *d*_0_/*d*_6_-labeled CDCA-3-G exhibited two pairs of diagnostic fragments (*m*/*z* 247.1294/253.1665 and *m*/*z* 229.1188/235.1559) and three common characteristic fragments (*m*/*z* 202.0739, 184.0634, and 166.0531) in MS/MS spectra (Figure 3A), while the *d*_0_/*d*_6_-labeled CDCA failed to produce these fragment ions (Figure 3B). These results demonstrate that the above-mentioned characteristic fragmentation patterns are unique to glucuronide derivatives, excluding other carboxyl-containing metabolites.

### Dual-Filtering Strategy

**Figure 4:**
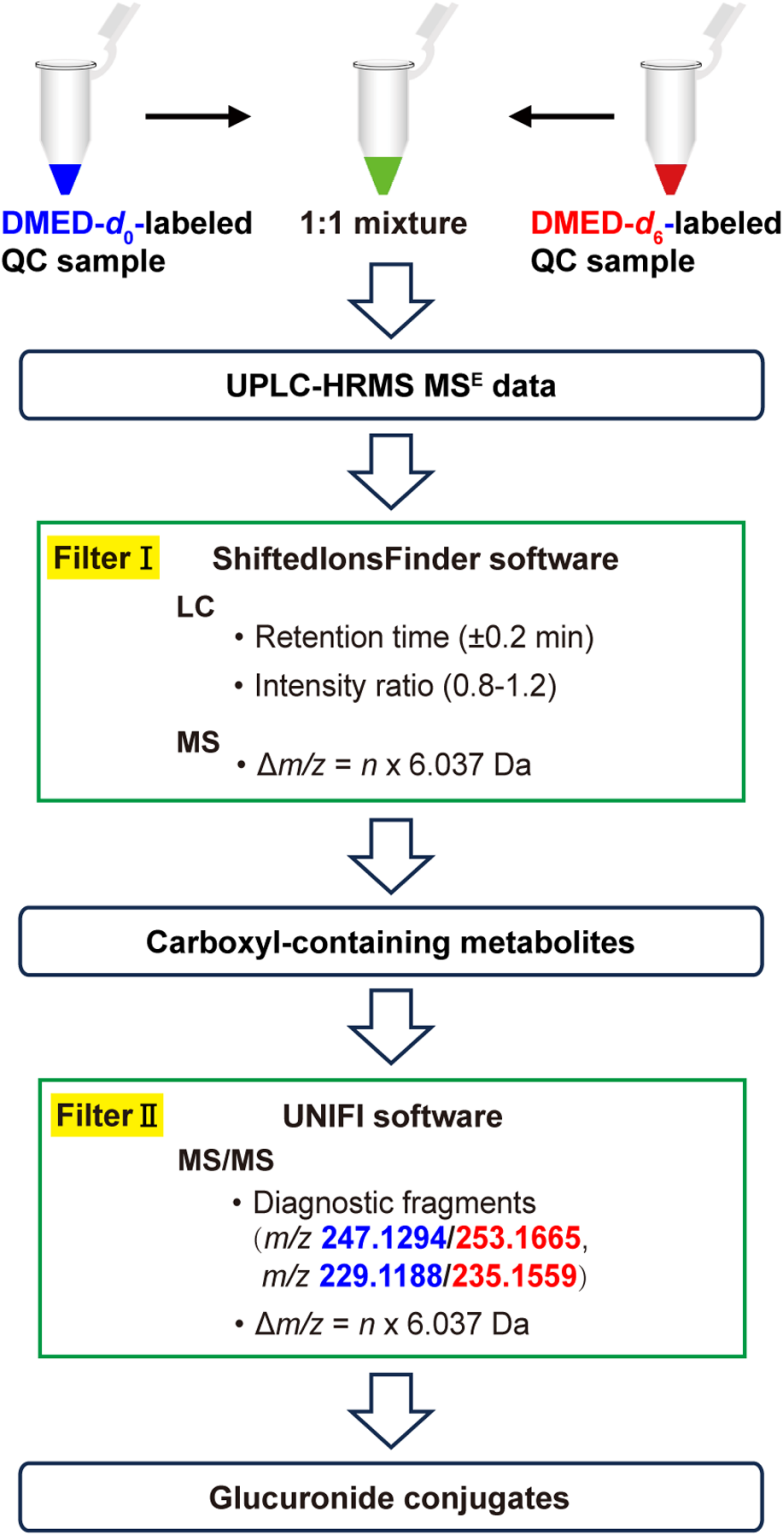
Workflow of dual-filtering strategy for screening DMED-labeled glucuronide metabolites.

Based on the unique fragmentation patterns observed in glucuronide derivatives, we proposed a dual-filtering strategy for comprehensive and confident profiling of glucuronate-conjugated metabolites in biological samples. As depicted in Figure 4, the light- and heavy-labeled QC samples are mixed in equal proportions before LC–MS analysis. Carboxyl-containing metabolites, including glucuronides, were screened using filter I. This involves identifying peak pairs with similar retention times, similar peak intensities, and a characteristic mass difference. To specifically highlight glucuronide metabolites, the characteristics of the labeled glucuronides in their MS/MS spectra, i.e., two pairs of diagnostic ions originating from the cleavage of the glycosidic bond (DMED-labeled glucuronic acid group) and dehydration of the derivatized glucuronic acid group, were employed as filter II. Specifically, MS^E^ raw data were acquired in the positive-ion mode by UPLC-QTOF MS and processed by Progenesis QI for alignment, peak picking, and exporting a feature list. The resulting peak list, which included accurate *m*/*z*, RT, fragment ion count, and intensity, was imported into ShiftedIonsFinder program, a standalone Java tool developed by Suzuki’s group,^31^ to screen DMED-*d*_0_ and DMED-*d*_6_-generated isotope peak pairs with similar retention times (±0.2 min), a peak intensity ratio of approximately 1:1 (0.8–1.2), and an *m*/*z* difference of *n* × 6.037 Da. Herein, “*n*” indicates the number of carboxyl groups rather than the glucuronic acid group. The n value can be calculated by dividing the mass difference between DMED-*d*_6_- and DMED-*d*_0_-labeled molecular ion peaks in the MS spectra by 6.037 Da (Figure S8). The resulting ions were recognized as carboxyl-containing metabolite candidates. Subsequently, the discovery module of the UNIFI Scientific Information System (Waters Corp., MA, USA) was utilized to screen the metabolites containing fragment ion pairs with *m*/*z* 247.1294/253.1665 in their MS/MS spectra with 20 ppm of mass tolerance, which resulted from the loss of the deconjugated aglycone from DMED*-d*_0_/DMED*-d*_6_-labeled metabolites. The screened metabolites were further filtered with fragment ions at 229.1188/235.1559, generated by subsequent dehydration. The potential glucuronate-conjugated metabolites could be confidently and effectively highlighted by following these procedures.

### Urinary Glucuronide Submetabolome Profiling with QC Samples

**Figure 5:**
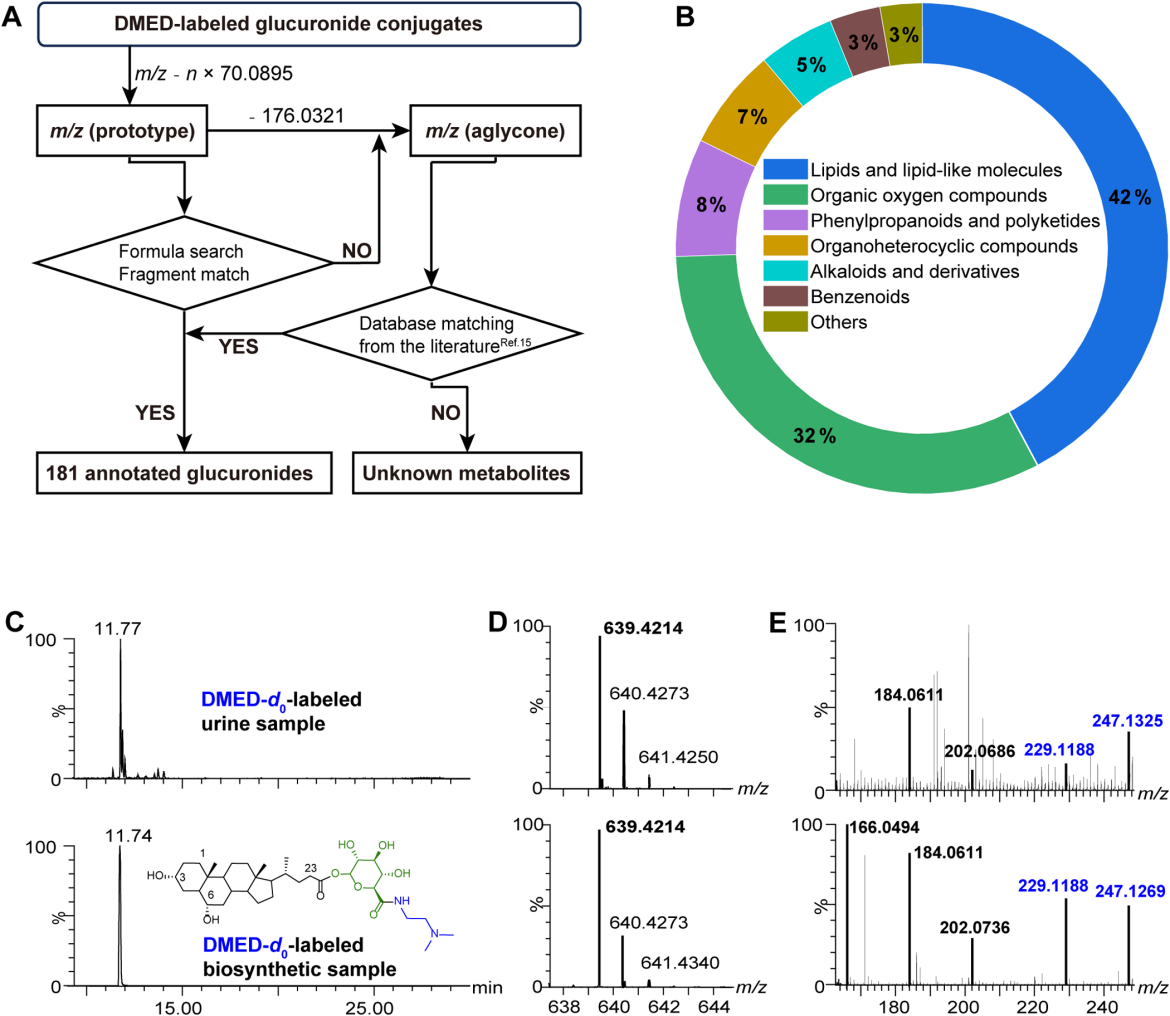
(A) Workflow for the annotation of glucuronides and (B) chemical classification of annotated glucuronides. (C–E) Biosynthesis-aided identification. Comparison of (C) chromatographic peak, (D) MS spectra, and (E) MS/MS spectra from urinary metabolites and biosynthetic hyodeoxycholic acid 24-glucuronide.

The dual-filtering strategy was applied to profile urinary glucuronides in QC samples pooled from 20 CRC patients (Figure S9). A total of 1735 carboxylated metabolites were identified after applying filter I, among which 685 metabolites were finally determined to be glucuronides with filter II (Table S2), with the structures of three unambiguously identified using commercial standards. However, the structural information for most glucuronides is unavailable in databases and commercial standards, presenting significant challenges in their structural annotation. We have proposed an identification workflow to address this issue, as illustrated in Figure 5A. Initially, the mass of the DMED-labeled glucuronide, which bears the *N*,*N*′-dimethyl ethylenediamine group, was subtracted by *n* × (C_4_H_12_N_2_·H_2_O) (*n* × *m*/*z* 70.0895, where n represents the number of carboxyl groups), resulting in the accurate mass of the corresponding native glucuronides. When *n* = 1, the carboxyl group is exclusively associated with the glucuronic group, indicating that the metabolite is a monoglucuronide. For *n* ≥ 2, the degree of glucuronidation (mono-, di-, or multiglucuronide) can be determined by examining the number of neutral losses of C_10_H_18_N_2_O_5_/C_10_H_12_D_6_N_2_O_5_ (246.1216/252.1592 Da) based on the MS/MS spectra of DMED-*d*_0_/*d*_6_-labeled glucuronides. Taking CDCA-3-G, a monoglucuronide with two carboxyl groups, as an example, the fragments derived from the labeled aglycone (*m*/*z* 463.3900/469.4276) resulting from a single neutral loss were exclusively identified, confirming that the conjugate is a monoglucuronide (Figure 2B). Similar findings were observed in the MS/MS spectra of glycyrrhizin, a diglucuronide with three carboxyl groups. The carboxyl count (*n* = 3) was determined by rounding [(1051.7861–1033.6751)/6.037], suggesting that a maximum of three glucuronic acids are conjugated (Figure S8C). Fragments derived from one neutral loss (*m*/*z* 787.5585/799.6299) and two neutral losses (*m*/*z* 541.4369/547.4746) were readily observed, while no fragments were generated from experiencing a third neutral loss (Figure 2D). Moreover, based on the number of carboxyl and glucuronic acid groups, the identification of these native glucuronides was performed by searching in biochemical databases, including HMDB^32^ (https://hmdb.ca/), PubChem^33^ (https://pubchem.ncbi.nlm.nih.gov/), and ChemSpider (http://www.chemspider.com/) databases with an MS match tolerance of 20 ppm. Furthermore, the accurate mass of the corresponding aglycones was obtained by subtracting the mass of the glucuronic acid group (C_6_H_8_O_6_, *m*/*z* = 176.0321 Da). In a study by Chen Y. C, et al.,^15^ a PDMS2E-DIA-based hydrolysis strategy was employed to identify 211 aglycones that could potentially undergo glucuronidation. In this study, these aglycones with calculated accurate mass were annotated by comparing their MS data with the database containing information about the 211 corresponding unconjugated metabolites. This approach was adopted to enhance the confidence level of the annotations, given the considerable likelihood of numerous aglycones undergoing glucuronidation.

Using the proposed workflow, the structures of 181 glucuronate-conjugated metabolites were tentatively assigned. These metabolites were then structurally classified into 7 groups using the ClassyFire system, a web-based application for automated structural classification of chemical entities (Figure 5B and Table S3). The predominant classes among the identified glucuronides were lipids and lipid-like molecules (76, 42%), organic oxygen compounds (58, 32%), and phenylpropanoids and polyketides (14, 8%). Similar to the results of the database search (Figure S3), lipids and lipid-like molecules were represented by endogenous steroid hormones (e.g., estradiol glucuronide and androsterone glucuronide) and bile acids [e.g., cholic acid (CA) glucuronide and deoxycholic acid glucuronide]. This distribution suggests that our strategy can effectively detect structurally diverse glucuronide conjugates.

Biosynthesis of glucuronide was conducted using a human liver microsome to validate the reliability of the structural annotation using the workflow. For instance, the ion *m*/*z* 639.4214 detected at RT 11.77 min was putatively annotated as hyodeoxycholic acid 24-glucuronide (HDCA-24-G, DMED-*d*_0_-labeled) with the workflow. Due to a lack of commercial standard, HDCA-24-G was biosynthesized from the glucuronidation reaction of HDCA in human liver microsomes (Figure S10) and served as a standard for comparison of the RT, MS spectra, and characteristic fragments of MS/MS spectra with those of the putatively identified glucuronide (Figure 5C–E). This biosynthesis demonstrated the reliability of our strategy and provided a viable alternative for the comprehensive identification of glucuronides in situations in which commercial standards are unavailable.

### Urinary Glucuronides Separate Early and Advanced-Stage CRC Patients

**Figure 6:**
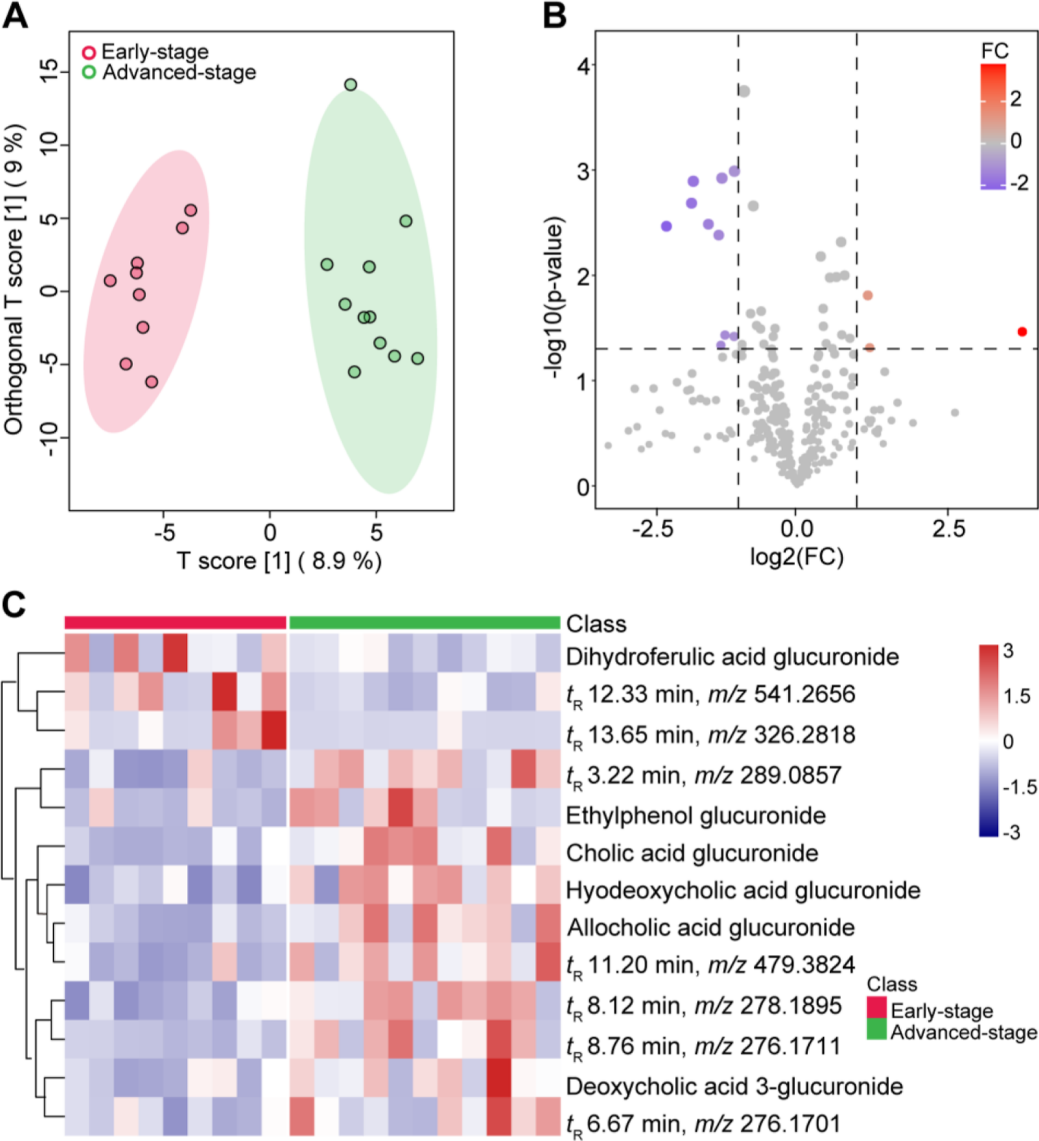
(A) OPLS-DA score plot and the (B) corresponding volcano plot based on glucuronide contents in urine samples from CRC patients at different stages. (C) Heat map visualizing the variations of differential glucuronides between the early stage and advanced stage of CRC.

Urinary glucuronide profiles were analyzed in urine samples from 20 patients at different CRC stages using DMED-*d*_0_ derivatization. The OPLS-DA demonstrated a clear separation between the early and advanced stages based on their urinary glucuronide profiles (*R*^2^*X* = 0.09, *R*^2^*Y* = 0.85, and *Q*^2^ = 0.45) (Figure 6A). Thirteen differentiatial glucuronides (FC > 2, VIP > 1 and *P* < 0.05) were highlighted by the volcano plot (Figure 6B), and six of them were successfully annotated. Among annotated differential glucuronides, four bile acid glucuronides, including one primary bile acid glucuronide (CA glucuronide) and three secondary bile acid glucuronides (hyodeoxycholic acid glucuronide, allocholic acid glucuronide, and deoxycholic acid 3-glucuronide), were significantly higher in patients with advanced CRC when compared to those in the early stage (Figure 6C). Bile acid homeostasis plays a crucial role in the etiology and pathogenesis of CRC.^34,35^ Primary bile acids, such as CA, are excreted into the intestine and then converted to secondary bile acids by gut microbes. Glucuronidation is one of the main biotransformation pathways for bile acids, accounting for 12–36% of total bile acid excretion in urine.^36^ Secondary bile acids, particularly deoxycholic acid (DCA), have been reported to promote CRC development.^37^ Studies have reported significantly higher fecal DCA levels in CRC patients compared to healthy individuals, and patients with high DCA levels have shown a higher risk of recurrence of large adenomas after colorectal tumor resection surgery.^38^ Analysis of bile acids in tumor tissues from 228 CRC patients using LC–MS revealed elevated levels of 12 bile acids, including CA and DCA, in right-sided colon tumors compared to left-sided colon tumors.^39^ These findings suggest the potential of these glucuronides, particularly bile acid glucuronides, as biomarkers for CRC diagnosis. However, further research with a larger population is needed to validate these findings. Additionally, two dietary-derived microbial metabolites, ethylphenol (EP) glucuronide and dihydroferulic acid (DFA) glucuronide, were highlighted. EP glucuronide was enriched, while DFA glucuronide exhibited a decrease in the advanced CRC (Figure S11). Further research is necessary to elucidate the exact role and significance of these glucuronides in CRC.

## Conclusions

This study established a chemical isotope labeling and dual-filtering strategy for comprehensively profiling glucuronide conjugates in biological samples using a pair of isotopically labeled reagents, DMED*-d*_0_ and DMED*-d*_6_. By leveraging the characteristic fragmentation patterns of the labeled glucuronides, potential glucuronide conjugates could be sensitively detected, rapidly screened, and efficiently annotated. Using the developed strategy, a total of 685 potential glucuronide conjugates were screened in urine samples from CRC patients. Through the integration of database search and fragmentation pattern analysis, we successfully annotated 181 glucuronide conjugates. Notably, several differential glucuronides, including bile acid glucuronides, were identified as potential markers for distinguishing the early stage and the advanced-stage CRC. Our proposed strategy demonstrates substantial promise for comprehensive profiling of the glucuronide metabolome, offering broad coverage and robust confidence in detecting changes associated with CRC and other diseases.

## Supporting Information Available

Chemical structures of 15 glucuronide standards; LC chromatograms and MS spectra of DMED-*d*_6_; ^1^H NMR spectra of DMED-*d*_0_ and DMED-*d*_6_; statistics for glucuronide conjugates and classification of endogenous glucuronides in the HMDB and PubChem; optimization of reaction temperature, time, and the ratio of DMED to substrate; extracted ion chromatograms of four representative DMED-labeled glucuronides using two organic modifiers; impacts of injection volume on chromatographic separation of DMED-labeled glucuronides; optimization of CV and CE values; chemical structure, chromatographic peak, and MS spectra of DMED-*d*_0_- and DMED-*d*_6_-labeled 3 representative glucuronides; base peak chromatograms of DMED-labeled and mixed urine samples; biosynthetic reaction of glucuronide; and abundance of six annotated differential glucuronides between the two groups (PDF)
Clinical and biochemical characteristics of CRC patients, potential candidates of glucuronide metabolites screened from pooled urine samples, and detailed information on annotated glucuronides (XLSX)

## References

[ref1] Lampou V. K.; Poller B.; Huth F.; Fischer A.; Kullak-Ublick G. A.; Arand M.; Schadt H. S.; Camenisch G. Novel insights into bile acid detoxification via CYP, UGT and SULT enzymes. Toxicol. In Vitro 2023, 87, 105533 10.1016/j.tiv.2022.105533. 36473578

[ref2] Barua A. B.; Sidell N. Retinoyl β-Glucuronide: A Biologically Active Interesting Retinoid. J. Nutr. 2004, 134, 286S–289S. 10.1093/jn/134.1.286S. 14704335

[ref3] Van Vleet T. R.; Liu H.; Lee A.; Blomme E. A. Acyl glucuronide metabolites: Implications for drug safety assessment. Toxicol. Lett. 2017, 272, 1–7. 10.1016/j.toxlet.2017.03.003. 28286018

[ref4] Yue T.; Chen R.; Chen D.; Liu J.; Xie K.; Dai J. Enzymatic Synthesis of Bioactive *O*-Glucuronides Using Plant Glucuronosyltransferases. J. Agric. Food Chem. 2019, 67 (22), 6275–6284. 10.1021/acs.jafc.9b01769. 31083910

[ref5] Kciuk M.; Marciniak B.; Kontek R. Irinotecan—Still an Important Player in Cancer Chemotherapy: A Comprehensive Overview. Int. J. Mol. Sci. 2020, 21 (14), 4919 10.3390/ijms21144919. 32664667 PMC7404108

[ref6] Beger R. D.; Schmidt M. A.; Kaddurah D. R. Current Concepts in Pharmacometabolomics, Biomarker Discovery, and Precision Medicine. Metabolites 2020, 10 (4), 129 10.3390/metabo10040129. 32230776 PMC7241083

[ref7] Chen J.; Zhang X.; Cao R.; Lu X.; Zhao S.; Fekete A.; Huang Q.; Schmitt-Kopplin P.; Wang Y.; Xu Z.; et al. Serum 27-nor-5β-Cholestane-3,7,12,24,25 Pentol Glucuronide Discovered by Metabolomics as Potential Diagnostic Biomarker for Epithelium Ovarian Cancer. J. Proteome Res. 2011, 10 (5), 2625–2632. 10.1021/pr200173q. 21456628

[ref8] Globisch D.; Eubanks L. M.; Shirey R. J.; Pfarr K. M.; Wanji S.; Debrah A. Y.; Hoerauf A.; Janda K. D. Validation of onchocerciasis biomarker N -acetyltyramine- O -glucuronide (NATOG). Bioorg. Med. Chem. Lett. 2017, 27 (15), 3436–3440. 10.1016/j.bmcl.2017.05.082. 28600214 PMC5510726

[ref9] Vaz F. M.; Bootsma A. H.; Kulik W.; Verrips A.; Wevers R. A.; Schielen P. C.; DeBarber A. E.; Huidekoper H. H. A newborn screening method for cerebrotendinous xanthomatosis using bile alcohol glucuronides and metabolite ratios. J. Lipid Res. 2017, 58 (5), 1002–1007. 10.1194/jlr.P075051. 28314860 PMC5408618

[ref10] Camilleri P.; Buch A.; Soldo B.; Hutt A. J. The influence of physicochemical properties on the reactivity and stability of acyl glucuronides. Xenobiotica 2018, 48 (9), 958–972. 10.1080/00498254.2017.1384967. 28967291

[ref11] Mostarda S.; Passeri D.; Carotti A.; Cerra B.; Colliva C.; Benicchi T.; Macchiarulo A.; Pellicciari R.; Gioiello A. Synthesis, physicochemical properties, and biological activity of bile acids 3-glucuronides: Novel insights into bile acid signalling and detoxification. Eur. J. Med. Chem. 2018, 144, 349–358. 10.1016/j.ejmech.2017.12.034. 29275233

[ref12] Yuan L.; Sophia Xu X.; Ji Q. C. Challenges and Recommendations in Developing LC–MS/MS Bioanalytical Assays of Labile Glucuronides and Parent Compounds in the Presence of Glucuronide Metabolites. Bioanalysis 2020, 12 (9), 615–624. 10.4155/bio-2020-0055. 32441529

[ref13] Fabregat A.; Pozo O. J.; Marcos J.; Segura J.; Ventura R. Use of LC-MS/MS for the Open Detection of Steroid Metabolites Conjugated with Glucuronic Acid. Anal. Chem. 2013, 85 (10), 5005–5014. 10.1021/ac4001749. 23586472

[ref14] Yan Z.; Li T.; Wei B.; Wang P.; Wan J.; Wang Y.; Yan R. High-resolution MS/MS metabolomics by data-independent acquisition reveals urinary metabolic alteration in experimental colitis. Metabolomics 2019, 15, 70 10.1007/s11306-019-1534-1. 31041724

[ref15] Chen Y. C.; Wu H. Y.; Chang C. W.; Liao P. C. Post-Deconvolution MS/MS Spectra Extraction with Data-Independent Acquisition for Comprehensive Profiling of Urinary Glucuronide-Conjugated Metabolome. Anal. Chem. 2022, 94 (6), 2740–2748. 10.1021/acs.analchem.1c03557. 35119834

[ref16] Qi B. L.; Liu P.; Wang Q. Y.; Cai W. J.; Yuan B. F.; Feng Y. Q. Derivatization for liquid chromatography-mass spectrometry. TrAC, Trends Anal. Chem. 2014, 59, 121–132. 10.1016/j.trac.2014.03.013.

[ref17] Zhang Q. F.; Xiao H. M.; Zhan J. T.; Yuan B. F.; Feng Y. Q. Simultaneous determination of indole metabolites of tryptophan in rat feces by chemical labeling assisted liquid chromatography-tandem mass spectrometry. Chin. Chem. Lett. 2022, 33 (11), 4746–4749. 10.1016/j.cclet.2022.01.004.

[ref18] Tao W. B.; Xie N. B.; Cheng Q. Y.; Feng Y. Q.; Yuan B. F. Sensitive determination of inosine RNA modification in single cell by chemical derivatization coupled with mass spectrometry analysis. Chin. Chem. Lett. 2023, 34 (10), 108243 10.1016/j.cclet.2023.108243.

[ref19] Yang R. J.; Zou J.; Liu J. Y.; Dai J. K.; Wan J. B. Click chemistry-based enrichment strategy for tracing cellular fatty acid metabolism by LC-MS/MS. J. Pharm. Anal. 2023, 13 (10), 1221–1231. 10.1016/j.jpha.2023.05.001. 38024853 PMC10657974

[ref20] Matsumoto T.; Yamazaki W.; Jo A.; Ogawa S.; Mitamura K.; Ikegawa S.; Higashi T. A Method for Quantification of Tetrahydroglucocorticoid Glucuronides in Human Urine by LC/MS/MS with Isotope-coded Derivatization. Anal. Sci. 2018, 34 (9), 1003–1009. 10.2116/analsci.18SCP02. 29887546

[ref21] Niyonsaba E.; Easton M. W.; Feng E.; Yu Z.; Zhang Z.; Sheng H.; Kong J.; Easterling L. F.; Milton J.; Chobanian H. R.; et al. Differentiation of Deprotonated Acyl-*N*-and *O*-Glucuronide Drug Metabolites by Using Tandem Mass Spectrometry Based on Gas-Phase Ion–Molecule Reactions Followed by Collision-Activated Dissociation. Anal. Chem. 2019, 91 (17), 11388–11396. 10.1021/acs.analchem.9b02717. 31381321

[ref22] Guo Y.; Shah A.; Oh E.; Chowdhury S. K.; Zhu X. Determination of Acyl-*O-*, and *N-*Glucuronide Using Chemical Derivatization Coupled with Liquid Chromatography–High-Resolution Mass Spectrometry. Drug Metab. Dispos. 2022, 50 (5), 716–724. 10.1124/dmd.122.000832. 35241454

[ref23] Costa A. R.; de Oliveira M. L.; Cruz I.; Gonçalves I.; Cascalheira J. F.; Santos C. R. The Sex Bias of Cancer. Trends Endocrinol. Metab. 2020, 31 (10), 785–799. 10.1016/j.tem.2020.07.002. 32900596

[ref24] He Y.; Luo Y.; Chen H.; Chen J.; Fu Y.; Hou H.; Hu Q. Profiling of carboxyl-containing metabolites in smokers and non-smokers by stable isotope labeling combined with LC-MS/MS. Anal. Biochem. 2019, 569, 1–9. 10.1016/j.ab.2018.12.006. 30543805

[ref25] Zhu Q. F.; Zhang T. Y.; Qin L. L.; Li X. M.; Zheng S. J.; Feng Y. Q. Method to Calculate the Retention Index in Hydrophilic Interaction Liquid Chromatography Using Normal Fatty Acid Derivatives as Calibrants. Anal. Chem. 2019, 91 (9), 6057–6063. 10.1021/acs.analchem.9b00598. 30943013

[ref26] Zheng S. J.; Liu S. J.; Zhu Q. F.; Guo N.; Wang Y. L.; Yuan B. F.; Feng Y. Q. Establishment of Liquid Chromatography Retention Index Based on Chemical Labeling for Metabolomic Analysis. Anal. Chem. 2018, 90 (14), 8412–8420. 10.1021/acs.analchem.8b00901. 29924596

[ref27] Yuan B. F.; Zhu Q. F.; Guo N.; Zheng S. J.; Wang Y. L.; Wang J.; Xu J.; Liu S. J.; He K.; Hu T.; Zheng Y. W.; Xu F. Q.; Feng Y. Q. Comprehensive Profiling of Fecal Metabolome of Mice by Integrated Chemical Isotope Labeling-Mass Spectrometry Analysis. Anal. Chem. 2018, 90 (5), 3512–3520. 10.1021/acs.analchem.7b05355. 29406693

[ref28] Zhu Q. F.; Yan J. W.; Zhang T. Y.; Xiao H. M.; Feng Y. Q. Comprehensive Screening and Identification of Fatty Acid Esters of Hydroxy Fatty Acids in Plant Tissues by Chemical Isotope Labeling-Assisted Liquid Chromatography–Mass Spectrometry. Anal. Chem. 2018, 90 (16), 10056–10063. 10.1021/acs.analchem.8b02839. 30052436

[ref29] Xia F. B.; Wan J. B. Chemical derivatization strategy for mass spectrometry-based lipidomics. Mass Spectrom. Rev. 2023, 42 (1), 432–452. 10.1002/mas.21729. 34486155

[ref30] Johnson D.; Boyes B.; Orlando R. J. The Use of Ammonium Formate as a Mobile-Phase Modifier for LC-MS/MS Analysis of Tryptic Digests. Biomol. Technol. 2013, 24 (4), 187–197. 10.7171/jbt.13-2404-005. PMC3825588 24294112

[ref31] Kera K.; Ogata Y.; Ara T.; Nagashima Y.; Shimada N.; Sakurai N.; Shibata D.; Suzuki H. ShiftedIonsFinder: A standalone Java tool for finding peaks with specified mass differences by comparing mass spectra of isotope-labeled and unlabeled data sets. Plant Biotechnol. 2014, 31 (3), 269–274. 10.5511/plantbiotechnology.14.0609c.

[ref32] Wishart D. S.; Guo A.; Oler E.; Wang F.; Anjum A.; Peters H.; Dizon R.; Sayeeda Z.; Tian S.; Lee B. L.; et al. HMDB 5.0: the Human Metabolome Database for 2022. Nucleic Acids Res. 2022, 50 (D1), D622–D631. 10.1093/nar/gkab1062. 34986597 PMC8728138

[ref33] Kim S.; Chen J.; Cheng T.; Gindulyte A.; He J.; He S.; Li Q.; Shoemaker B. A.; Thiessen P. A.; Yu B.; et al. PubChem 2023 update. Nucleic Acids Res. 2023, 51 (D1), D1373–D1380. 10.1093/nar/gkac956. 36305812 PMC9825602

[ref34] Ocvirk S.; O’Keefe S. J. Dietary fat, bile acid metabolism and colorectal cancer. Semin. Cancer Biol. 2021, 73, 347–355. 10.1016/j.semcancer.2020.10.003. 33069873

[ref35] Caliceti C.; Punzo A.; Silla A.; Simoni P.; Roda G.; Hrelia S. New Insights into Bile Acids Related Signaling Pathways in the Onset of Colorectal Cancer. Nutrients 2022, 14 (14), 2964 10.3390/nu14142964. 35889921 PMC9317521

[ref36] Almé B.; Sjövall J. Analysis of bile acid glucuronides in urine. Identification of 3α,6α,12α-trihydroxy-5β-cholanoic acid. Steroid Biochem. 1980, 13 (8), 907–916. 10.1016/0022-4731(80)90164-8. 7464137

[ref37] Liu Y.; Zhang S.; Zhou W.; Hu D.; Xu H.; Ji G. Secondary Bile Acids and Tumorigenesis in Colorectal Cancer. Front. Oncol. 2022, 12, 813745 10.3389/fonc.2022.813745. 35574393 PMC9097900

[ref38] Kawano A.; Ishikawa H.; Kamano T.; Kanoh M.; Sakamoto K.; Nakamura T.; Otani T.; Sakai T.; Kono K. Significance of fecal deoxycholic acid concentration for colorectal tumor enlargement. Asian Pac. J. Cancer Prev. 2010, 11 (6), 1541–1546. 21338194

[ref39] Cai Y.; Shen X.; Lu L.; Yan H.; Huang H.; Gaule P.; Muca E.; Theriot C. M.; Rattray Z.; Rattray N. J.; et al. Bile acid distributions, sex-specificity, and prognosis in colorectal cancer. Biol. Sex Differ. 2022, 13 (1), 61 10.1186/s13293-022-00473-9. 36274154 PMC9590160

