# Supplementary material for: Chemical Isotope Labeling and Dual-Filtering Strategy for Comprehensive Profiling of Urinary Glucuronide Conjugates
Source: Anal Chem. 2024 Aug 5;96(33):13576–87. doi: 10.1021/acs.analchem.4c02339 (PMC11339728; doi:10.1021/acs.analchem.4c02339)
Supplement: Supplementary file 1 — ac4c02339_si_001.pdf [file ac4c02339_si_001.pdf]

## Supporting Information

# Chemical Isotope Labeling and Dual-filtering Strategy for Comprehensive Profiling of Urinary Glucuronide Conjugates

Zhi-Qiang Chen[‡, a], Ru-Jie Yang[‡, a], Chao-Wei Zhu[b], Yang Li[b], Ru Yan[a, *], Jian-Bo Wan[a, *]

[a] State Key Laboratory of Quality Research in Chinese Medicine, Institute of Chinese Medical Sciences, University of Macau, Taipa, Macao SAR, China

[b] Shenzhen People's Hospital, Shenzhen, Guangdong, China

[‡] These authors contributed equally to this work.

***Correspondences:**

**Prof. Jian-Bo Wan,**

E-mail: jbwan@um.edu.mo

Room 6034, Building N22, Institute of Chinese Medical Sciences,

University of Macau, Avenida da Universidade, Taipa, Macao SAR, China

**Prof. Ru Yan,**

E-mail: ruyan@um.edu.mo@um.edu.mo

Room 7011, Building N22, Institute of Chinese Medical Sciences,

University of Macau, Avenida da Universidade, Taipa, Macao SAR, China

# Table of Content

**Figure S1.** Chemical structures of 15 glucuronide standards........................................................S3

**Figure S2.** (**A**) LC chromatograms and (**B**) MS spectra of DMED-$d_6$, (**C**) $^1$H NMR spectra of DMED-$d_0$ (up) and DMED-$d_6$ (down) ..........................................................................................S4

**Figure S3.** Statistics for glucuronide conjugates and classification of endogenous glucuronides in the HMDB and PubChem.........................................................................................................S5

**Figure S4.** Optimization of derivatization conditions, including (**A**) reaction temperature, (**B**) reaction duration and (**C**) the ratio of DMED to substrate...................................................................S5

**Figure S5.** Extracted ion chromatograms (EICs) of four representative DMED-labeled glucuronides using (**A**) ammonium formate and (**B**) formic acid as organic modifier...........................................S6

**Figure S6.** The impacts of injection volume on chromatographic separation of DMED-labeled glucuronides.................................................................................................................S7

**Figure S7.** Optimization of (**A**) cone voltage and (**B**) collision energy values. ...............................S8

**Figure S8.** Chemical structure (left), chromatographic peak (middle), and MS spectra (right) of DMED-$d_0$- (blue) and DMED-$d_6$- (red) labeled (**A**) melatonin glucuronide, (**B**) thyroxine glucuronide, and (**C**) glycyrrhizin.............................................................................................................S8

**Figure S9.** Base peak chromatograms of DMED-labeled and mixed urine samples........................S9

**Figure S10.** Biosynthetic reaction of glucuronide.............................................................................S9

**Figure S11.** Abundance of six annotated differential glucuronides between the two groups of early-stage and advanced-stage.....................................................................................................S10

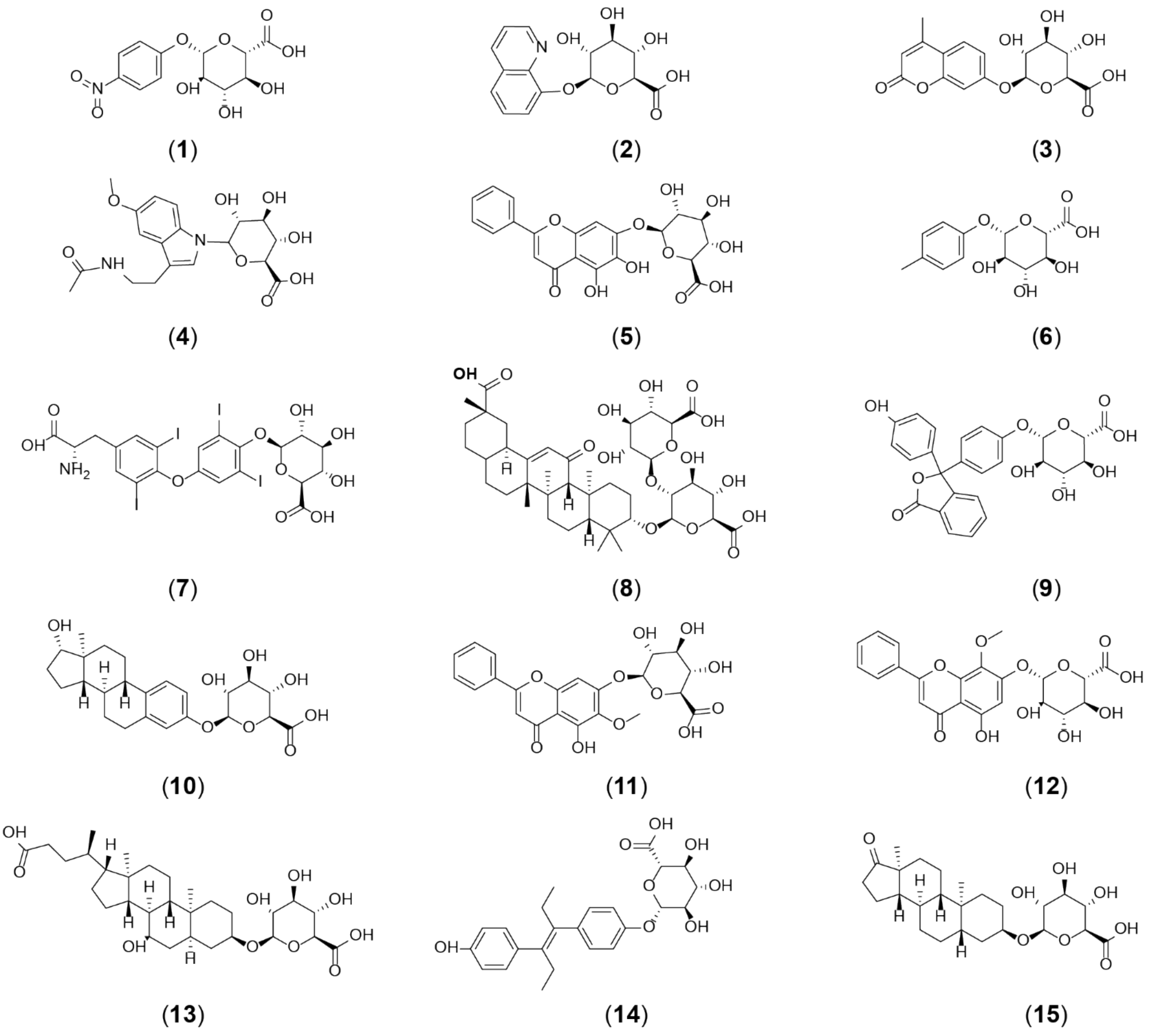


**Figure S1.** Chemical structures of 15 glucuronide standards.

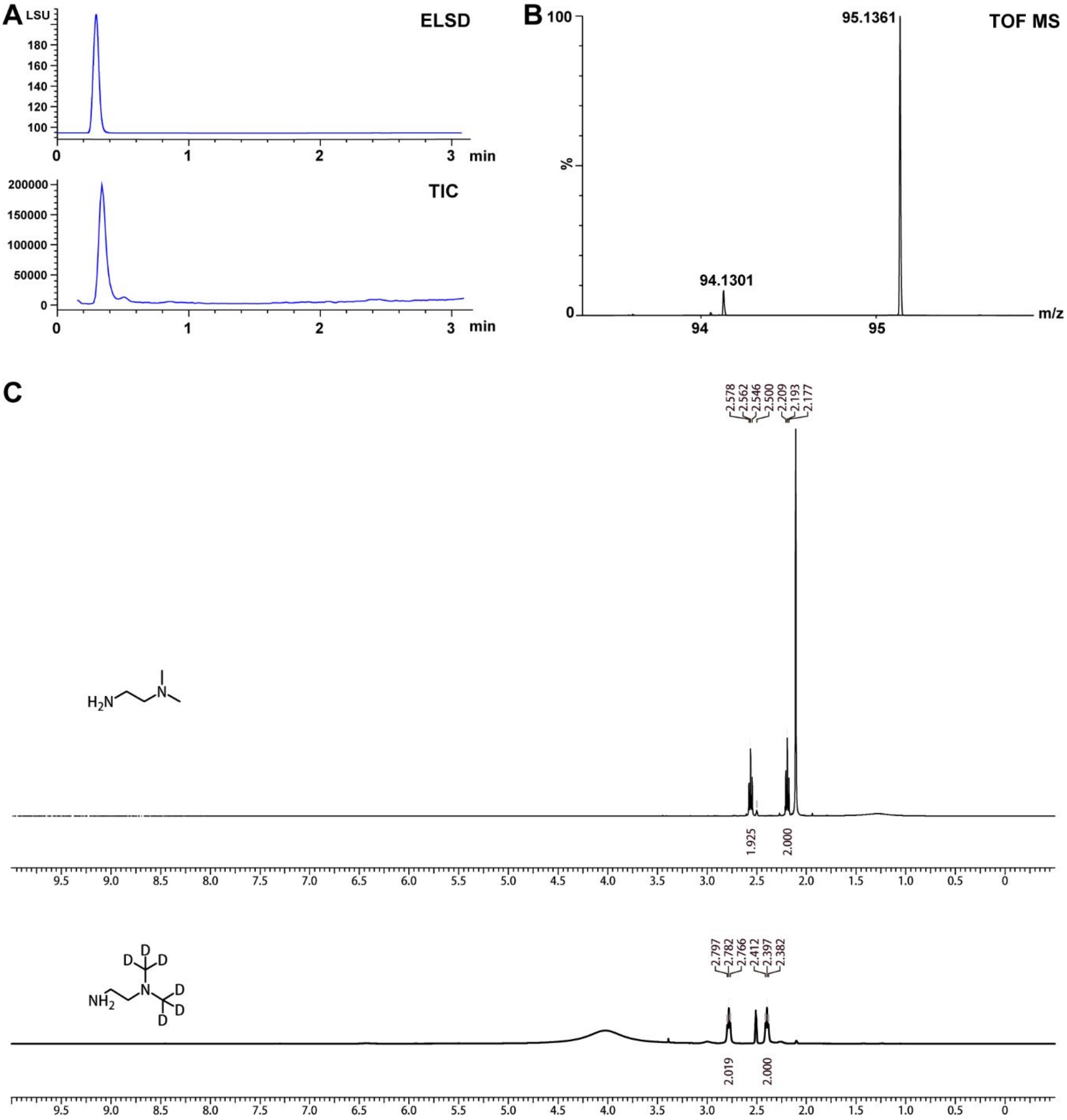


**Figure S2.** (**A**) LC chromatograms and (**B**) MS spectra of DMED-$d_6$, (**C**) $^1$H NMR spectra of DMED-$d_0$ (up) and DMED-$d_6$ (down). DMED, N, N-dimethyl ethylenediamine.

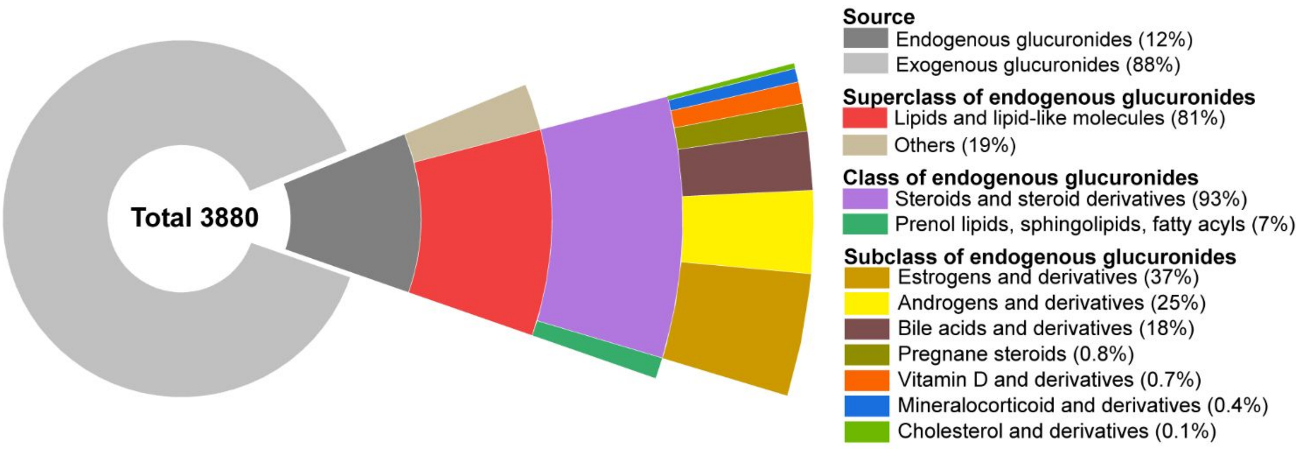


**Figure S3.** Statistics for glucuronide conjugates and classification of endogenous glucuronides in the HMDB and PubChem.

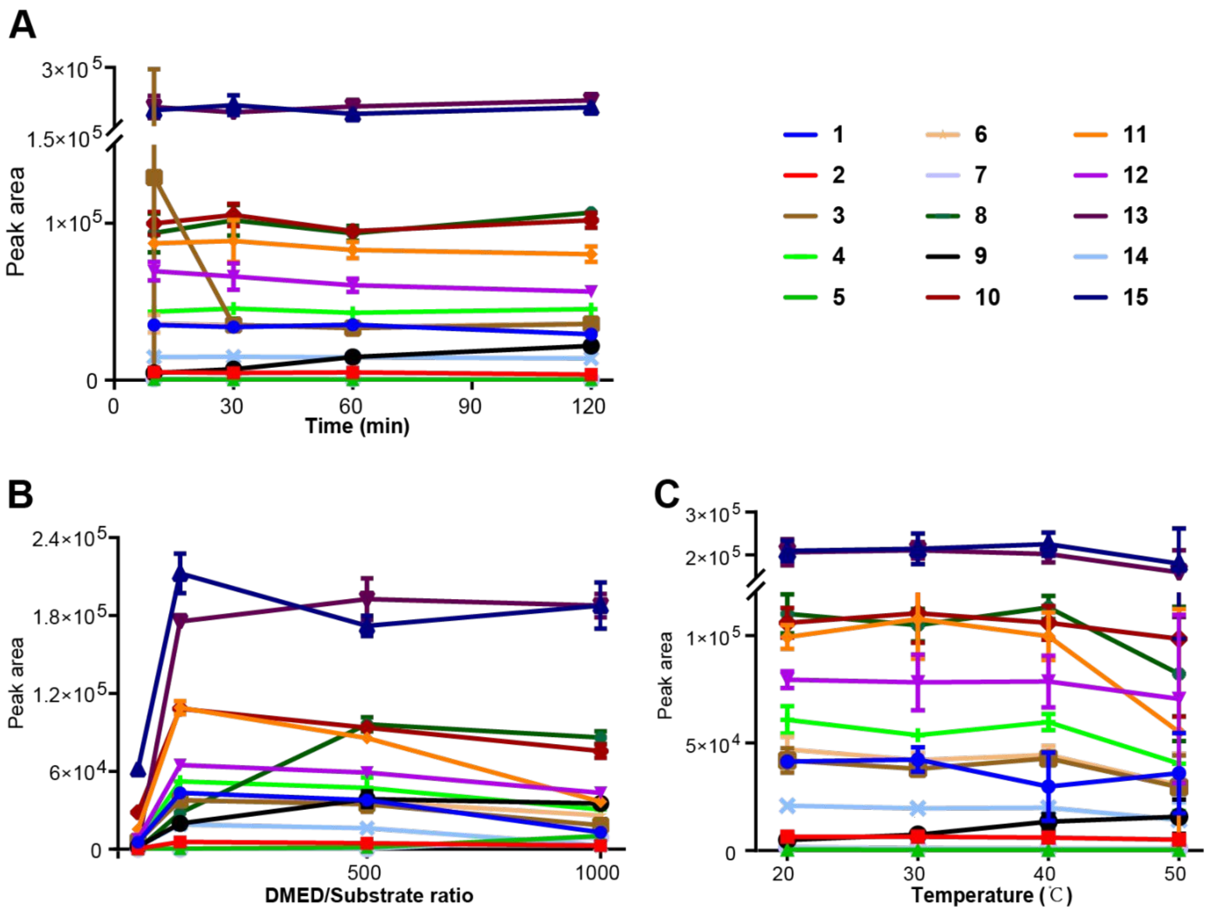


**Figure S4.** Optimization of derivatization conditions, including (**A**) reaction temperature, (**B**) reaction duration and (**C**) the ratio of DMED to substrate. The number of glucuronide standards is represented in the same manner as **Table 1**.

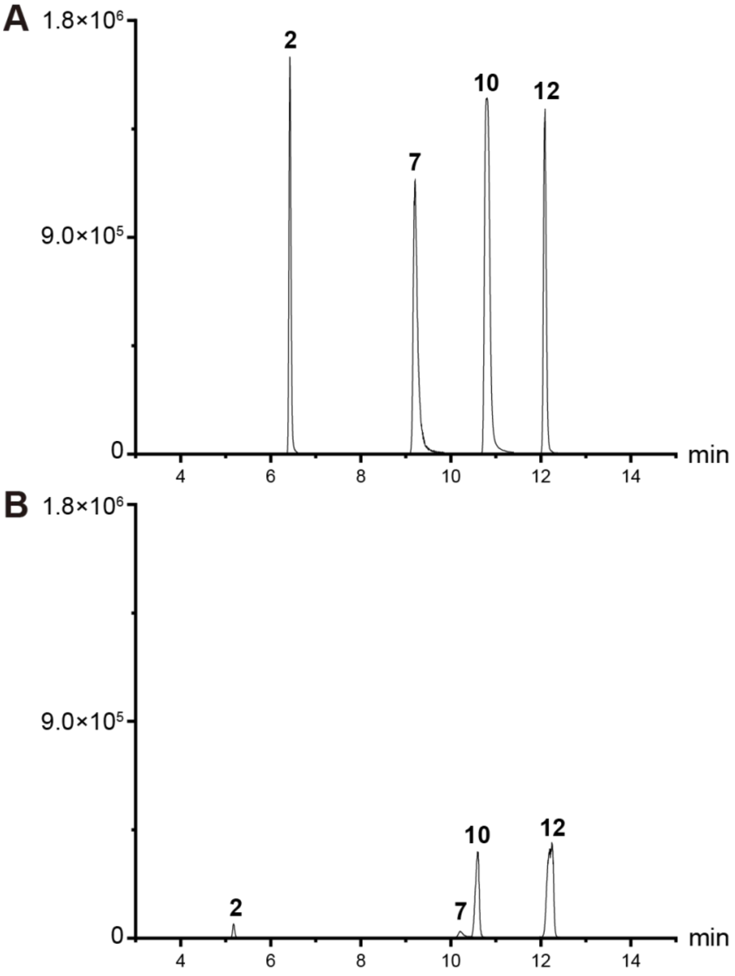


**Figure S5.** Extracted ion chromatograms (EICs) of four representative DMED-labeled glucuronides using (**A**) ammonium formate and (**B**) formic acid as organic modifier. **2**, 8-hydroxyquinoline glucuronide; **7**, thyroxine glucuronide; **10**, estradiol 3-glucuronide; **12**, wogonoside.

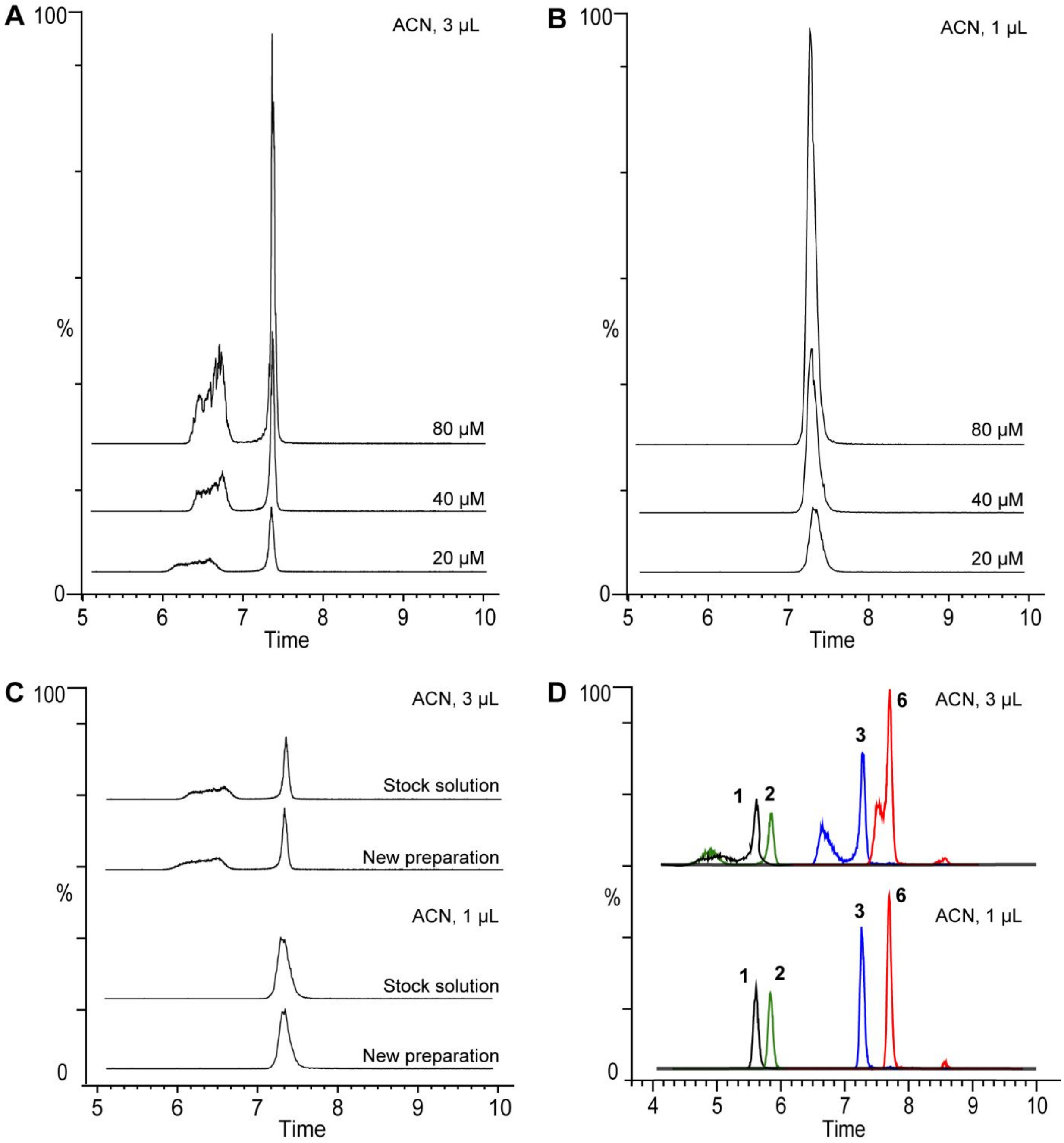


**Figure S6.** The impacts of injection volume on chromatographic separation of DMED-labeled glucuronides. EICs of 4-methylumbelliferyl glucuronide at different concentrations with injection volumes of (**A**) 3 µL and (**B**) 1 µL. (**C**) EICs of 4-methylumbelliferyl glucuronide using stock and freshly prepared solutions with injection volumes of 3 µL and 1 µL. (**D**) EICs of four DMED-labeled glucuronides at different injection volumes. **1**, 4-nitrophenyl glucuronide; **2**, 8-hydroxyquinoline glucuronide; **3**, 4-methylumbelliferyl glucuronide; **6**, p-Cresol glucuronide.

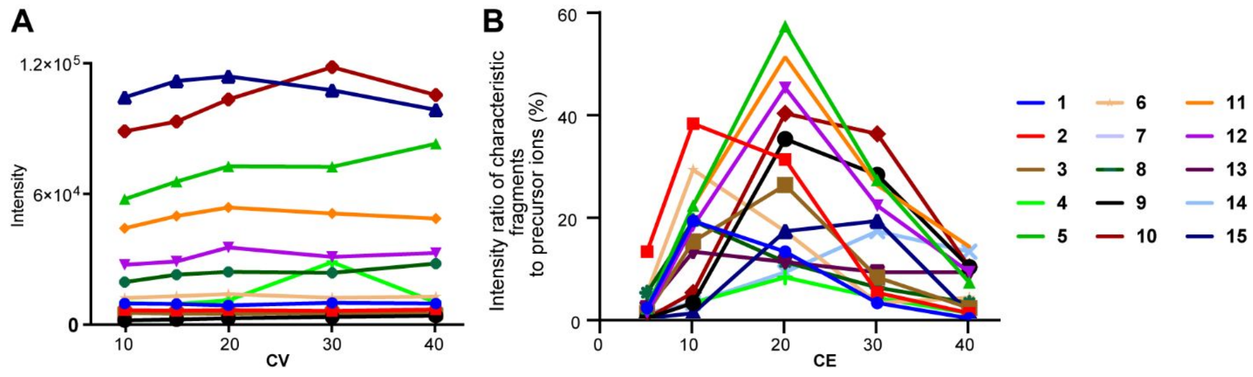


**Figure S7.** Optimization of (**A**) cone voltage and (**B**) collision energy values. The number of glucuronide standards is represented in the same manner as **Table 1**.

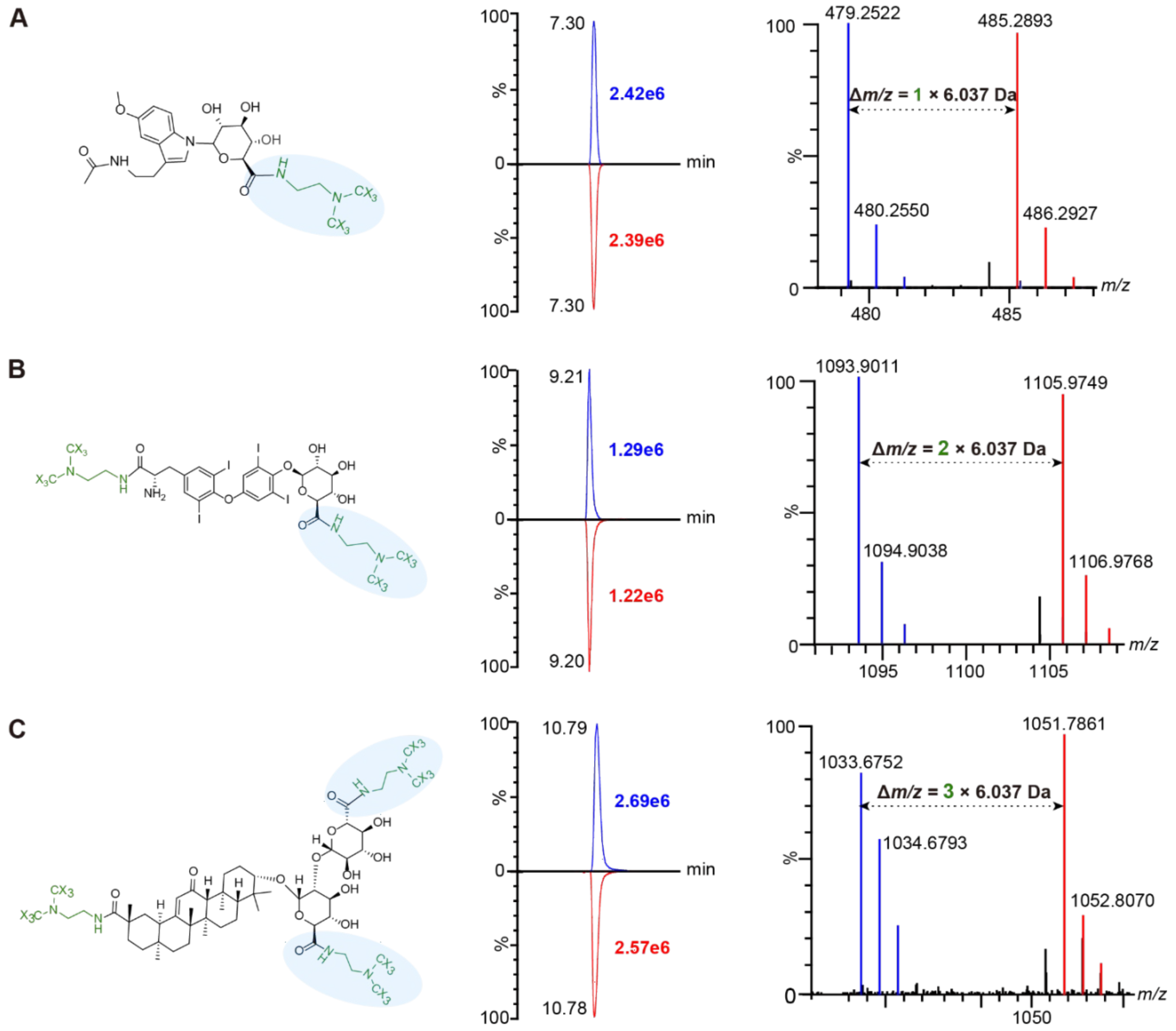


**Figure S8.** Chemical structure (left), chromatographic peak (middle), and MS spectra (right) of DMED-$d_0$- (blue) and DMED-$d_6$- (red) labeled (**A**) melatonin glucuronide, (**B**) thyroxine glucuronide, and (**C**) glycyrrhizin.

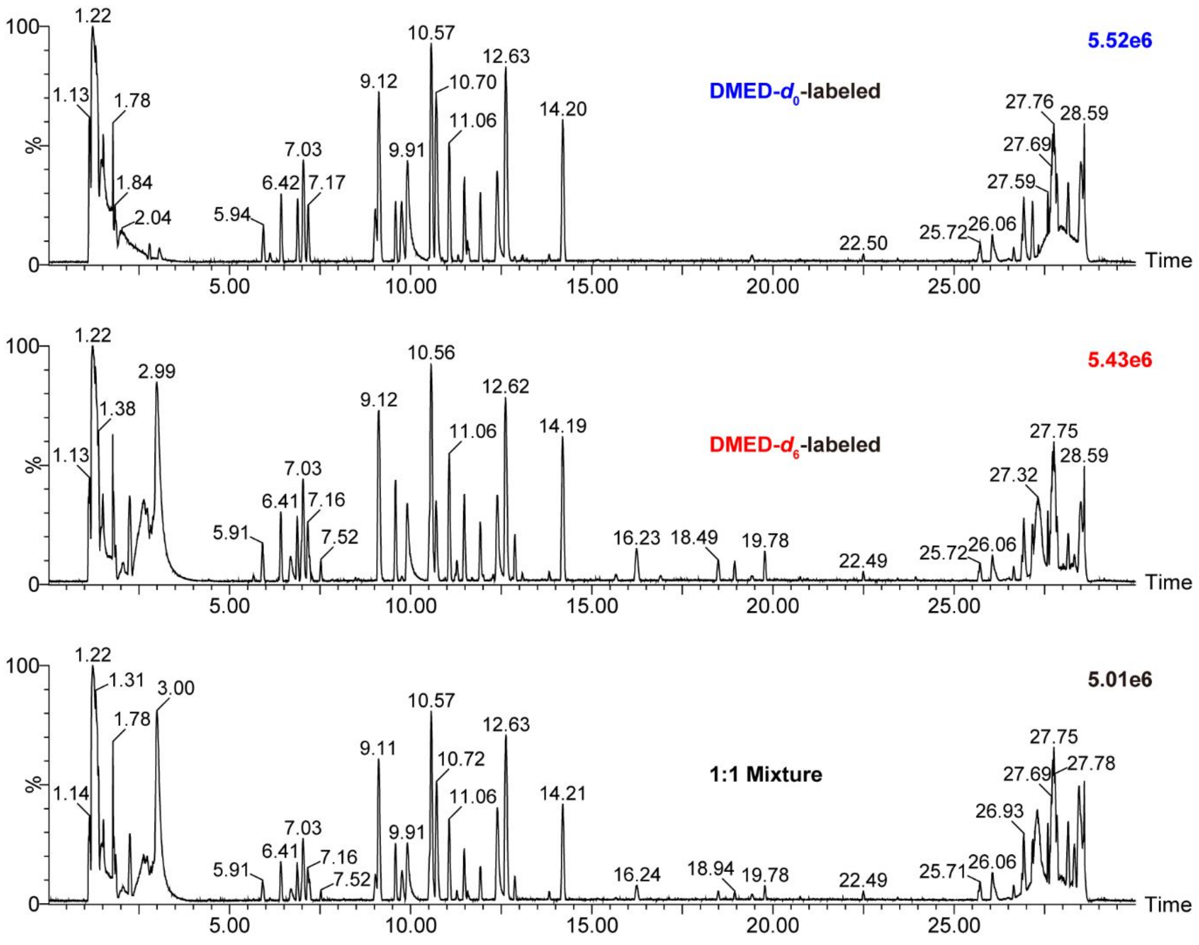


**Figure S9.** Base peak chromatograms of DMED-labeled and mixed urine samples.

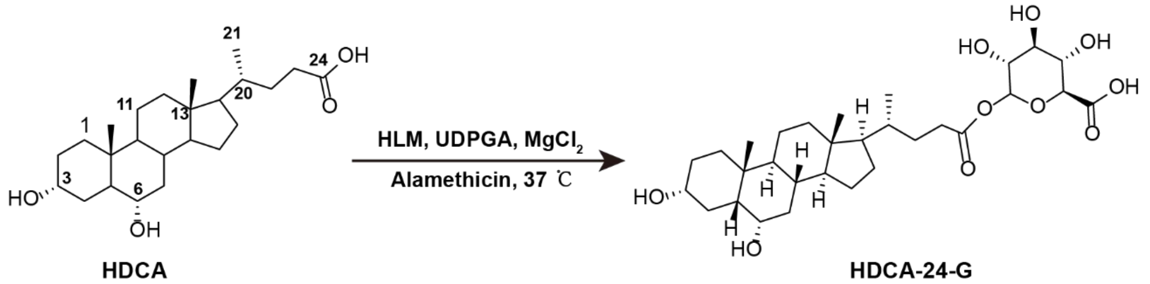


**Figure S10.** Biosynthetic reaction of glucuronide. HDCA, hyodeoxycholic acid; HDCA-24-G, hyodeoxycholic acid 24-glucuronide; HLM, human liver microsome; UDPGA, uridine diphosphate glucuronic acid.

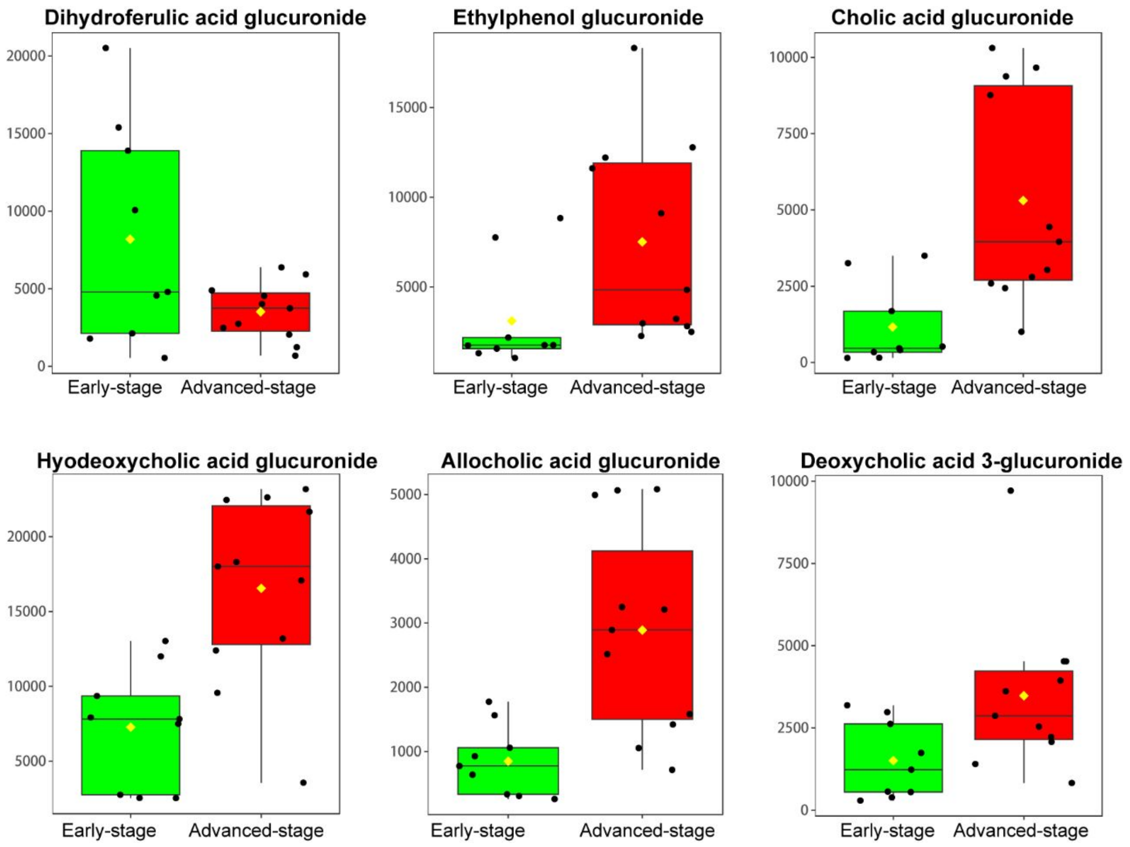


**Figure S11.** Abundance of six annotated differential glucuronides between the two groups of early-stage and advanced-stage.
